# Age-invariant genes: multi-tissue identification and characterization of murine reference genes

**DOI:** 10.18632/aging.206192

**Published:** 2025-01-27

**Authors:** John T. González, Kyra Thrush-Evensen, Margarita Meer, Morgan E. Levine, Albert T. Higgins-Chen

**Affiliations:** 1 Department of Pathology, Yale University School of Medicine, New Haven, CT 06519, USA; 2 Altos Labs, Institute of Computation, San Diego, CA 92114, USA; 3 Department of Psychiatry, Yale University School of Medicine, New Haven, CT 06519, USA

**Keywords:** housekeeping genes, organ aging, RT-qPCR normalization, stable expression, mammalian transcriptome

## Abstract

Studies of the aging transcriptome focus on genes that change with age. But what can we learn from age-invariant genes—those that remain unchanged throughout the aging process? These genes also have a practical application: they can serve as reference genes in expression studies. Reference genes have mostly been identified and validated in young organisms, and no systematic investigation has been done across the lifespan. Here, we build upon a common pipeline for identifying reference genes in RNA-seq datasets to identify age-invariant genes across seventeen C57BL/6 mouse tissues (brain, lung, bone marrow, muscle, white blood cells, heart, small intestine, kidney, liver, pancreas, skin, brown, gonadal, marrow, and subcutaneous adipose tissue) spanning 1 to 21+ months of age. We identify 9 pan-tissue age-invariant genes, and many tissue-specific age-invariant genes. These genes are stable across the lifespan and are validated in independent bulk RNA-seq datasets and RT-qPCR. Age-invariant genes have shorter transcripts and are enriched for CpG islands. Interestingly, pathway enrichment analysis for age-invariant genes identifies an overrepresentation of molecular functions associated with some, but not all, hallmarks of aging. Thus, even though hallmarks of aging typically involve change, select genes associated with these hallmarks resist age-related change. Finally, our analysis provides a list of murine tissues where classical reference genes are appropriate for application in aging studies. However, no classical reference gene is appropriate across all aging tissues. Instead, we provide novel tissue-specific and pan-tissue reference genes for assays utilizing gene normalization (RT-qPCR) that can be applied to mice across the lifespan.

## INTRODUCTION

Aging, the accumulation of cellular, molecular, and physiological alterations in an organism over time, increases the risk of dysfunction, chronic disease, and mortality [1]. The advent of next-generation sequencing and other high-throughput technologies has allowed for data-driven analyses to discover age-linked gene expression changes and dysregulation. However, little effort has been directed toward identifying and understanding age-invariant genes – those that remain unchanged throughout the aging process. The utility of such genes would be twofold: (1) they can be used as reference genes in quantitative assays, and (2) they may share molecular features that allow them to resist changes with age.

The transcriptome exhibits substantial remodeling during the aging process, and many of these changes may drive declines in cellular function. By employing bulk RNA-seq across 17 mouse tissues, Schaum et al. identified clusters of genes with similar age trajectories associated with the hallmarks of aging [2]. Gene clusters increasing in expression included immune and stress response genes, while those decreasing in expression included genes involved in the extracellular matrix, mitochondria, and protein folding [2]. A global decrease in gene expression occurs with aging, such that when comparing older animals to younger animals, differentially expressed genes tend towards downregulation [3]. For tissue-specific genes, a divergence or specialization of distinct cell types is observed during development. In contrast, aging has been associated with a loss of specificity in transcriptional profiles [4] and an increase in transcriptional noise (increased variance between individuals) [5–7]. Interestingly, genes subject to age-related change have been linked to specific features, including transcript length and association with CpG islands [8, 9].

Studying age-invariant genes that do not change their expression and remain stable throughout the aging process may uncover complementary aging mechanisms. The notion of invariant genes has been a focus of biomedical research for over 50 years, but their study has been confined to young organisms or cell line perturbations [10]. Due to their relative stability, invariant genes have been utilized as internal reference controls for gene expression assays. Initially coined as housekeeping genes, these invariant genes are constitutively expressed at high levels, are subject to low fluctuations, and are often essential for proper cellular function [10–12]. The changing definition of the term “housekeeping gene” led the Minimum Information for Publication of Quantitative Real-Time PCR Experiments (MIQE) guidelines to update the term used for normalization to reference genes (RGs) [13], and we will utilize this term. There is no absolute standard list of RGs. There are many commonly used RGs used throughout the literature, which we refer to here as classical RGs. Importantly, classical RGs such as glyceraldehyde-3-phosphate dehydrogenase (GAPDH), actin β (ACTB), and β2-microglobulin (B2M), have been found to be highly variable in certain contexts [11, 14]. Although an ultimate RG may not exist (consistent across all possible tissues, cell types, cell cycle stages, experimental conditions, diseases, and developmental phases), identifying invariant genes in specific contexts and sample types is possible and essential for proper experimental design [14, 15].

Little work has been done to identify and validate RGs that are stable throughout the aging process, i.e., age-invariant RGs. These genes would be invariant across the lifespan, either within any given tissue (tissue-specific) or across all tissues (pan-tissue). Aging is known to impact classical reference gene expression: a mouse study, for example, found that age, sex, and frailty explicitly alter the expression of a majority of classical RGs examined [11, 14, 16, 17]. Within the aging field, studies are restricted to RGs identified in other fields rather than using a novel, aging-focused analysis. The few available studies examining RGs in aging employ targeted RT-qPCR validation of some of the aforementioned classical transcripts and recommend different RGs based on the genes and the parameters included. For example, GUSB increased with age in mouse skeletal muscle, making it a poor RG in that context, but it was the best RG candidate in human peripheral blood mononuclear cells [16, 18–21]. Another salient example for aging is Cdkn1a/p21. Cdkn1a/p21 is sometimes utilized as a reference gene in RT-qPCR normalization literature, even though it simultaneously serves as a marker of cellular senescence–one of the major hallmarks of aging, which is defined by change over time [22–24]. However, a systematic analysis of many RGs across many tissues has not yet been reported, and it is unclear which classical RGs remain age-invariant in which tissues. Thus, there is a pressing need to identify both classical and novel RGs appropriate for aging studies.

We now have the tools and datasets to identify age-invariant RGs. The first iterations of reference genes, which compose a majority of popular RGs, were not experimentally determined but selected because they were detected in all tissues and assumed to have little variability [10, 25]. With the development of 21st-century microarray and next-generation sequencing technologies, this question can finally be tackled from a data-rich perspective [25]. RNA-seq datasets have been successfully used to experimentally identify RGs in healthy human tissue [10, 11], mammalian animal models [14, 26], non-mammalian organisms [27], disease conditions [28] and even single-cell populations [29]. The variables included in the datasets for these analyses determine the application constraints of the resulting RGs. Novel data-rich unsupervised techniques paired with next-generation sequencing data remain an untapped resource for identifying RGs for aging studies and more fully understanding the dynamics of transcriptional change (or lack thereof) with aging.

Here, we leverage published approaches for RG identification [10] with appropriate refinements and apply them to public bulk RNA-seq datasets with samples collected across the full lifespan to identify murine age-invariant genes. We identify tissues for each classical RGs where they remain age-invariant. We show that, unlike our age-invariant genes, no classical RG is suitable for aging studies across all tissues. Finally, we characterize features and functions of these age-invariant genes. Of note, we opted to focus on the subset of age-invariant genes that can also serve as RGs - those that are also relatively highly expressed - due to their practical applications.

## RESULTS

### Identification of candidate age-invariant genes from RNA-Seq data

**Figure 1:**
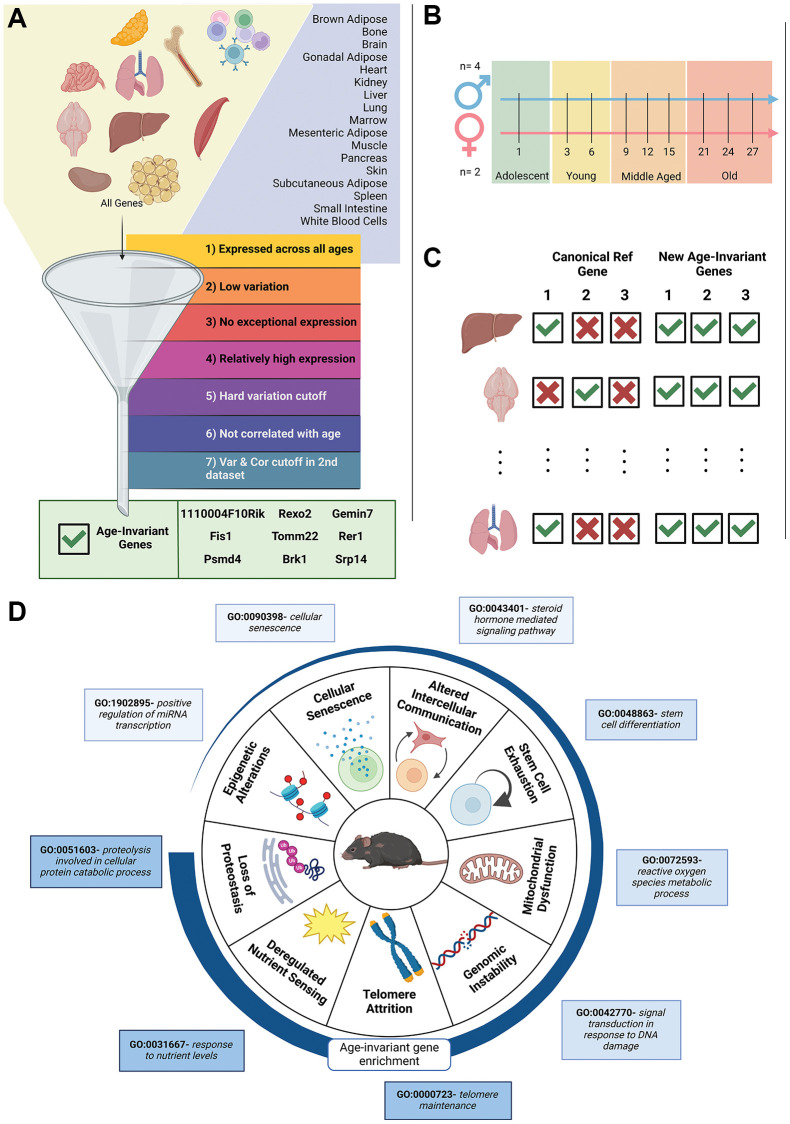
**Visual diagram of article contents.** (**A**) Bulk RNA-seq data from 17 murine tissues (GSE132040) were sequentially filtered through 7 criteria. The funnel is a visual depiction of the filtering process. Steps 1–4 are adapted from previous publications. We added criteria filters 5 and 6 to ensure low variation and no correlation with age. Criteria filter 7 was validation of low variation and no age correlation, performed in a second dataset for 11 of the 17 tissues. The filtering strategy resulted in 9 pan-tissue age-invariant genes (gene box). (**B**) Sample gender, age and life stage distributions of the samples in the dataset. A full table of samples can be found in Supplementary Table 10. (**C**) Classical reference genes are not applicable to all tissues in an aging context but age-invariant genes introduced here are. (**D**) Tissue aging-invariant genes are enriched to different extents for gene ontology terms associated with hallmarks of aging. Age-invariant genes have low enrichment in some (e.g. epigenetic alterations GO terms) and high enrichment in others (e.g. loss of proteostasis GO terms). Created with https://www.biorender.com/.

The design of our study to identify and characterize age-invariant genes is shown in Figure 1A–1D. Bulk RNA-seq data from the Tabula Muris Senis study [2] were utilized for age-invariant RG discovery. We analyzed 17 tissues: brown adipose tissue (BAT), bone, brain, gonadal adipose tissue (GAT), heart, kidney, limb, liver, lung, marrow, mesenteric adipose tissue (MAT), pancreas, subcutaneous adipose tissue (SCAT), skin, small intestine, spleen and white blood cells (WBCs) (Figure 1A). We performed quality control and only utilized samples where we could verify the tissue label (Supplementary Figure 1; Methods). The dataset contained female and male mice representing the 4 major lifespan stages: adolescent (1mo), young (3 and 6mo), middle-aged (9, 12, and 15mo), and old (21, 24, and 27mo) [30] (Figure 1B).

Tissues were independently analyzed by sequentially applying 7 filtering criteria through each tissue’s gene set (Figure 1A). Here, we utilize expression counts normalized to Transcripts Per Million (TPM) [17], which is similar to RT-qPCR as it approximates relative molar RNA concentration, as well as Trimmed Mean of M (TMM) [31], which leverages inter-sample information to reduce sensitivity to gene outliers. Both normalization techniques performed similarly well at identifying RGs in a recent systematic comparison of normalization methods [28]. Our approach leverages two different normalization techniques to reduce artifacts specific to individual methods. Each criterion, or filter, was applied to each tissue individually with both normalization methods; genes were only included in the tissue-filter gene list if they satisfied the requirement in both TPM and TMM normalized datasets.

Criteria are listed below, with exact details including equations in Methods. The filtering pipeline was applied to each tissue separately, with samples spanning the lifespan stages defined in Figure 1B. Thus, in the criteria below, “samples” refers to samples from the specific tissue that the filtering criteria is being applied to, rather than all samples across tissues. Although some genes have been identified as age-invariant within multiple tissues, this does not suggest they are invariant to tissue type and, thus should still be applied in a tissue-specific manner. Criteria 1–4 are adapted from an approach frequently used for RG identification from RNA-seq data [10, 27]:

1. Continuous expression: gene expressed in all samples.
2. Low variance: gene expression is similar for all samples (as assessed by standard deviation).
3. No exceptional expression/outliers: gene shows no outliers for any sample.
4. Medium to high gene expression: higher expression than the average gene for that tissue.

To ensure age-invariant gene list quality, we added three new filters to the identification criteria:

5. Low coefficient of variation (CV): gene expression is similar for all samples (as assessed by low variance relative to the mean).
6. No correlation between gene expression and age: expression is not significantly correlated with age.
7. External validation: Filters 5 and 6 were applied in publicly available validation datasets. Tissues with a validation dataset were BAT, brain, heart, kidney, muscle, liver, lung, SCAT, skin, small intestine, and WBCs (11 of 17 tissues).

**Figure 2:**
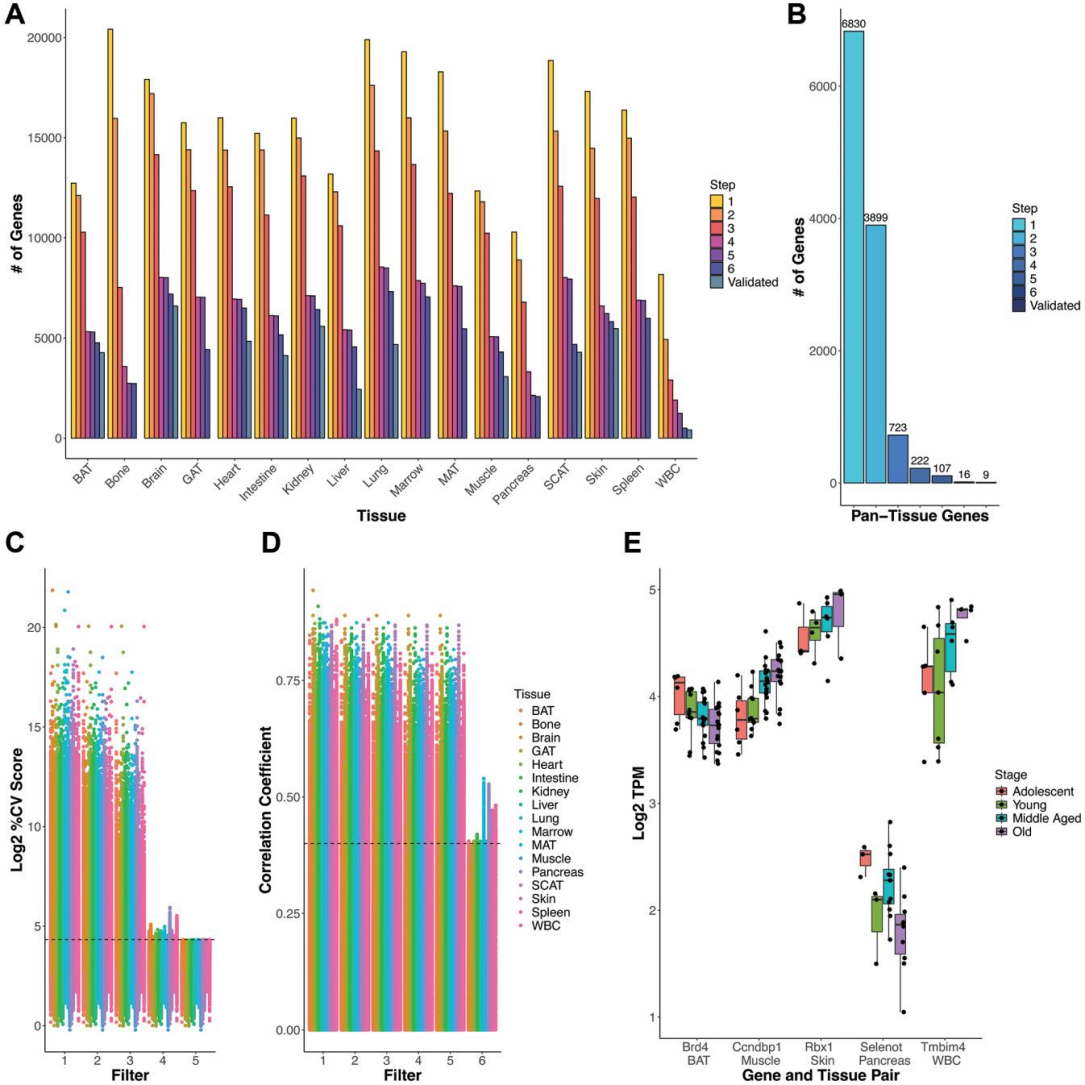
**Gene selection process and rationale.** (**A**) Gene count number remaining after each criteria/filter step for each tissue. (**B**) Count of genes present across all tissues at each step (e.g. pan-tissue) (**C**) % Coefficient of Variance (CV) for each gene calculated as SD/mean*100 distribution of log2 TPM gene expression values. Genes that satisfy every subsequent filter are plotted by the last filter applied. Filters 1–3 slowly decrease %CV and the cumulative effect of filters 1–4 generally results in a %CV of approximately 20% (marked by the dashed line). Filter 5 imposes a strict %CV <20% requirement for all tissue-gene pairs. (**D**) Age information must be included in exclusion criteria as low variation genes can still have a high correlation with age. Filter 6 (Spearman correlation *p*-value based removal) removes highly age-correlated genes. Dashed line corresponds to a correlation coefficient (y-axis) of 0.4, which for most tissues corresponds to a significant correlation with *p* = 0.05. Exact CV and age correlation information is found in Supplementary Table 2, in case readers wish to utilize other cutoffs in selecting RGs. (**E**) Log2 TPM (y-axis) values by life stage (color) for specific gene-tissue pairs (x-axis) for genes that satisfy filters 1–5, but are eliminated by filter 6. Boxplot line represents the group median while lower and upper limits of the boxplot correspond to the first (25%) and third (75%) quartiles.

The filters progressively refined the list of both tissue-specific (Figure 2A and Supplementary Table 1) and pan-tissue age-invariant genes (Figure 2B and Table 1). For reference, Supplementary Table 2 lists information on each gene’s %CV, slope with age, and correlation (coefficient and *p*-value) with age in each tissue, normalized in both TPM and TMM methods. Genes with a sample with no expression (i.e., 0) have empty statistics due to them being calculated on a log2 transformed scale. This table allows readers to select their own cutoffs if they choose. Supplementary Tables 3–9 contain lists of all genes that passed each consecutive filter in each tissue.

**Table 1:** MGI symbol and ID for our 9 pan-tissue age-invariant genes.

1110004F10Rik (MGI:1929274)	Fis1 (MGI:1913687)	Psmd4 (MGI:1201670)	Rexo2 (MGI:1888981)	Tomm22 (MGI:2450248)
Brk1 (MGI:1915406)	Gemin7 (MGI:1916981)	Rer1 (MGI:1915080)	Srp14 (MGI:107169)	

These genes were present across all tissues after all filtering steps and validation.

There were a few notable modifications to the original pipeline. First, we modified Criterion 4, which selects for relatively highly expressed genes and, therefore, is easily detected by RT-qPCR [10]. Because each tissue had different gene count distributions (for example brain, Supplementary Figure 2A vs. WBCs Supplementary Figure 2B), we deviated from the previous use of an arbitrary cutoff and employed an adjusted cutoff, removing genes with means below the mean of all genes expressed in a given tissue (log2 transformed) [27]. Consistent with previous publications [27], the cumulative effect of filters 2 (standard deviation cut-off) and 4 (mean cut-off) resulted in a percent coefficient of variation (%CV) of about 20% in most tissues (Figure 2C). However, given the lower average normalized gene expression in some tissues (Bone, Pancreas, Spleen, WBC), genes in these tissues surpassed this threshold. To ensure the genes obtained were truly low variance, we applied a hard cut-off of 20% CV (Filter 5). A %CV restriction provides the added benefit of limiting the variability stemming from any potential confounder — a variable that may change the expression levels of RGs—including frailty, sex, and age-related morbidities [16]. This approach combines Eisenberg et al.’s low variance definition of RGs and their alternative criterion of mid-to-high expression [10].

Second, we added Filter 6 to ensure age invariance. We had initially hypothesized that simply analyzing samples with a wide age range using the typical RG pipeline (filters 1–4) would be sufficient to filter out genes that change with age. Indeed, adding age groups to the analysis progressively discarded genes during the filtering process (Supplementary Figure 3A). This, however, could be due to the increase in samples (*n*) included in the analysis. To test whether the wide age range alone contributes important information, we applied the steps of the standard pipeline (filters 1–4) on samples belonging to only a particular lifespan stage and compared it to a cross-stage control with the same n. Including a wide range of ages by using cross-stage analysis discarded more genes compared to single-stage analysis for adolescent, middle-aged, and old stages (Supplementary Figure 3B). Surprisingly, this was not the case for the young adult stage (3-6mo old); we found this was likely due to a subset of genes that have high expression variability in young adults but are stable in other life stages (Supplementary Figure 3C–3F). We identified lists of genes that were age-invariant (with filters 1–4) only in young samples (Supplementary Figure 3C), age-invariant in young samples and other life stages (analyzed separately) (Supplementary Figure 3D), age-invariant in all life stages except young (Supplementary Figure 3E), and those age-invariant in the full dataset, i.e., all lifespan stages (Supplementary Figure 3F). All these lists revealed a similar pattern: some genes have higher variance (%CV) in young and old populations. The rightward shift in young and old samples reflect this. Within these high-variability stages, young samples have an overall higher proportion of high variance (over 20%CV) genes than old ones (Supplementary Figure 3C–3F). Furthermore, we found that some genes obtained through filters 1–5 still changed with age (Figure 2D, 2E). Thus, simply utilizing a wide age range in the typical pipeline does not necessarily help identify age-invariant genes. To address this finding, we added criterion 6, removing genes with statistically significant correlations with age for each tissue (Figure 2D).

Finally, to decrease the number of false positives, we validated the gene lists using a second bulk mRNA-seq dataset for 11 out of 17 tissues (except for bone, GAT, marrow, MAT, pancreas, and spleen). The number of validated genes is displayed in Figure 2A, 2B as Step 7. Specific counts and percentages can be found in Supplementary Table 1. For nearly all tissues, a supermajority (>70%) of candidate age-invariant genes were validated, except in the liver (54%) and lung (62%). The fewest number of age-invariant genes was observed in WBCs, possibly due to large changes in distributions of cell types over shorter timescales [32, 33] (Figure 2A).

### RT-qPCR validation of novel age-invariant reference genes

**Figure 3:**
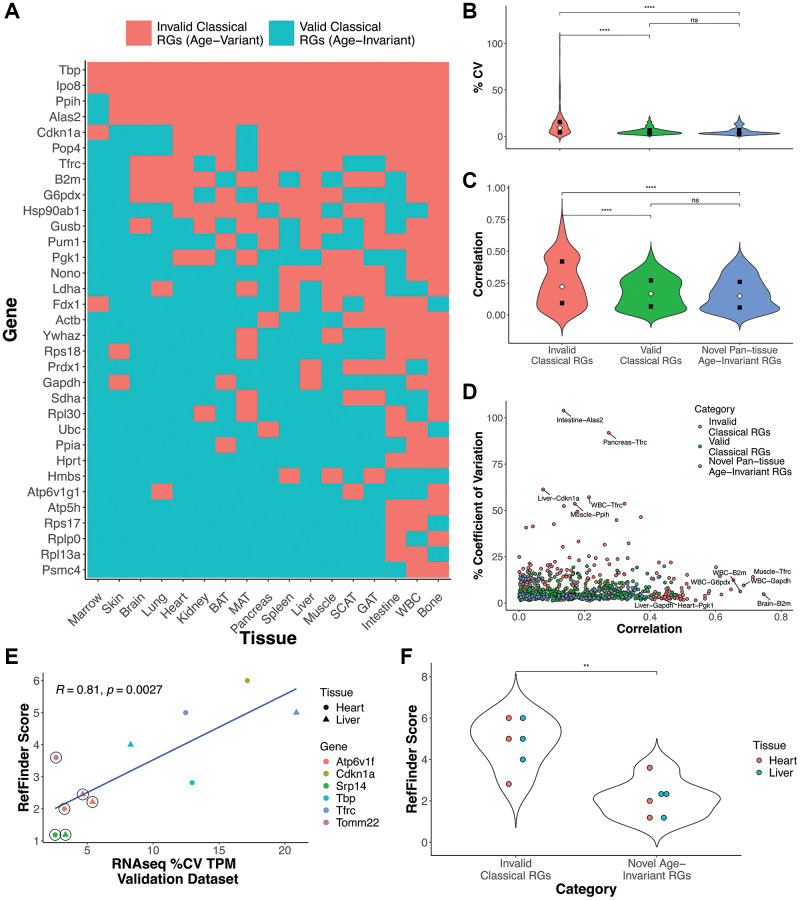
**Classical and novel RG performance in aging samples.** (**A**) Aging RG status of classical reference gene by tissue. Some tissue-RG pairs are age-invariant (blue), and are therefore good RG candidates for aging studies, while others did not satisfy our filtering criteria (red). (**B**, **C**) Coefficient of variance (**B**) or age correlation (**C**) violin plots of valid classical RGs (green), novel pan-tissue age-invariant RGs, and invalid classical RGs (red). (**D**) Scatterplot comparing %CV and age correlation for each tissue-RG pair. (**E**) Scatterplot comparing RT-qPCR Gene RefFinder score and mRNA-seq %CV in heart and liver. RefFinder and %CV scores were calculated from in-house and public validation datasets respectively. RefFinder is a summary score of BestKeeper, NormFinder, GeNorm and comparative delta-Ct values (analysis of these scores can be found in Supplementary Figure 5). Circled points indicate novel age-invariant RGs (Two pan-tissue: Tomm22 and Srp14; and one heart and liver age-invariant gene: Atp6v1f) while uncircled points specify classical RGs from Figure 3A. (**F**) Violin plots for RefFinder scores comparing invalid classical RGs and novel pan-tissue age-invariant RGs. Unless specified, *p*-values are obtained from a Welch Two Sample *t*-test (^**^p < 0.01, ^***^P < 0.0001, exact *p*-values in text).

Our analysis identified many tissue-specific age-invariant RGs (Supplementary Table 9). Many classical RGs are age-invariant in some tissues but not others (Figures 1C and 3A). Our list of classical RGs is compiled from the cited literature and BioRad’s PrimePCR Reference Gene panels. Thus, we propose a new list of 9 age-invariant genes common to all 17 tissues that can be used as reference genes in aging studies (Table 1). Classical RGs that failed our filtering process (invalid classical RGs) in a given tissue had higher coefficient of variation (Figure 3B) compared to classical RGs that passed our filters (valid classical RGs, *p* = 4.96e-14) and compared to our novel pan-tissue age-invariant genes (*p* = 1.04e-13). Invalid classical RGs also had a higher age correlation than valid classical RGs (*p* = 9.65e-08) and the novel pan-tissue age-invariant RGs (*p* = 1.13e-07) (Figure 3C). However, there was no significant difference between valid classical RG-tissue pairs and the novel pan-tissue age-invariant RGs based on these metrics, suggesting both are equally appropriate as reference genes within a given tissue. A direct comparison of age correlation with %CV shows that classical genes tend to fail on one of the two metrics (%CV or age correlation) but not both (Figure 3D). This finding highlights the need for the two novel filters we applied in the previous section in successfully finding age-invariant genes.

Our novel pan-tissue and tissue-specific (including valid classical RGs) age-invariant RGs can be utilized in the context of northern blot, RT-qPCR, and some RNA-seq normalization strategies in aging studies. Researchers have the choice of selecting from a tissue-specific gene list or from the nine pan-tissue genes. To validate this, independent heart and liver samples were used to generate RT-qPCR data for three age-invariant genes identified by our computational pipeline: Atp6v1f, Srp14, and Tomm22 (Supplementary Figure 4 and Figure 3E, 3F). Atp6v1f is a tissue-specific RG in the liver and heart, while Srp14 and Tomm22 are pan-tissue age-invariant genes. The novel samples consisted of mouse heart and liver samples in four categories: old (~19mo) female, old male, young (~8mo old) female, and young male. We compared these against three classical RGs that our analysis found to be invalid RGs in heart and liver: Cdkn1a, Tbp, and Tfrc. These classical reference genes generally had a wider cycle threshold distribution than the age-invariant genes, especially Tfrc and Cdkn1a (Supplementary Figure 4). Cdkn1a codes for cyclin-dependent kinase inhibitor 1A, also known as p21. Given that Cdkn1a is widely used as a marker of cell senescence [23], it is not surprising that it has a high degree of variability despite it being widely considered an RG in RT-qPCR normalization literature [18].

To assess gene RT-qPCR stability in the context of aging, we calculated the expression stability across multiple algorithms: BestKeeper [34] (Supplementary Figure 5A, 5B), geNorm [35] (Supplementary Figure 5C, 5D), NormFinde (Supplementary Figure 5E, 5F), and delta-CT method [36] (Supplementary Figure 5G, 5H). NormFinder is worth highlighting, as it takes into consideration both the inter- and intra-group variances (here the variation due to age and sex). These scores were utilized to calculate the summary RefFinder score (Figure 3E, 3F and Supplementary Figure 5I) [37]. The coefficient of variance for the discovery (Supplementary Figure 5A, 5C, 5E, 5G, 5I) and validation (Figure 3E and Supplementary Figure 5B, 5D, 5F, 5H) RNA-seq datasets strongly correlate with all stability algorithm values calculated on our in-house samples. For example, the coefficient of variance calculated in the validation RNA-seq dataset was correlated with the RefFinder score at a Pearson correlation = 0.81 (Figure 3E, *p*-value = 0.0027). This suggests that %CV from normalized RNA-seq samples could be used as an indicator of candidate reference genes for RT-qPCR experiments subject to the same conditions.

Classical RGs deemed to be invalid by our approach have significantly higher RefFinder qPCR scores (and are therefore worse RGs) than our novel age-invariant RGs (Figure 3F, *p*-value = 0.001799). This suggests pan-tissue age-invariant genes (Srp14 and Tomm22) or tissue-specific age-invariant genes (Atp6v1f in heart and liver) could be applied as part of normalization in age-related transcriptomic research. A combination of more than one of the age-invariant genes is recommended for RT-qPCR experiments, per the MIQE guidelines [13].

### Overlapping pathways for aging stable and aging dysregulated genes

**Figure 4:**
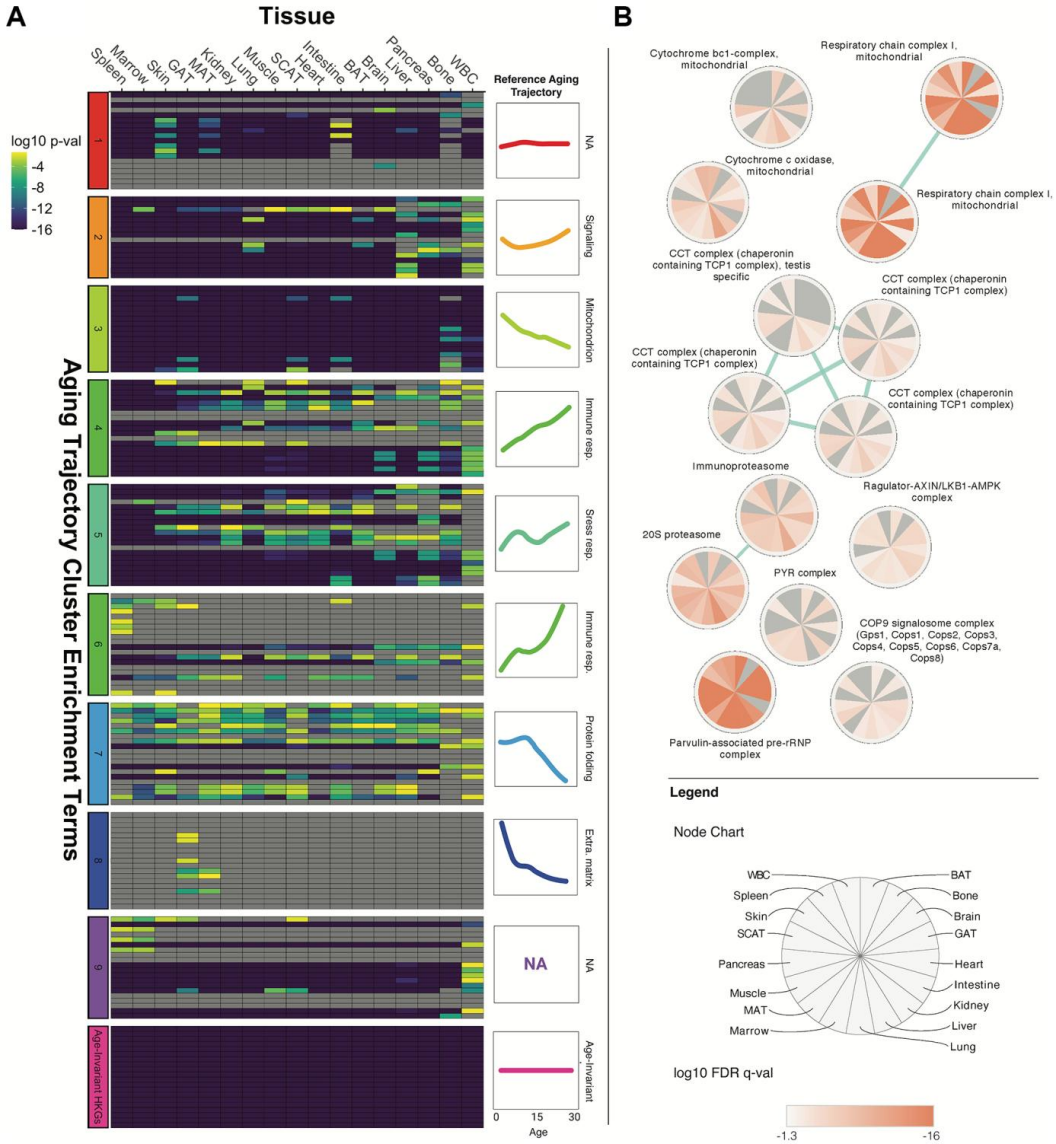
**Age-invariant genes are enriched for age-dysregulated gene functions.** (**A**) Tissue age-invariant genes are enriched for some of the same GO, KEGG and REACTOME terms that are also associated with clusters of age-dysregulated genes with linear and non-linear aging trajectories. Heatmap columns correspond to different tissues, while rows correspond to enrichment terms described in the dataset’s original publication by Schaum et al. [2]. The row clusters on the left of the heatmap and colored line-plots on the right of the heatmap correspond to 9 groups defined by Schaum et al. Age-invariant labels at the very bottom (pink) refer to age-invariant genes we identified for each tissue. Heatmap color corresponds to the Bonferroni-corrected *p*-value on a log10 scale from a Fisher's exact test quantifying enrichment. A version of this plot with term names can be found in Supplementary Figure 7A. An alternative analysis utilizing terms associated with hallmarks of aging found by Fraser et al. [39] can be found in Supplementary Figure 7B. (**B**) Tissue age-invariant genes are enriched for certain protein complexes. Each circular node corresponds to a protein complex, each slice within the node corresponds to a different tissue, and the coloration within slices reflects the log10 False Discovery Rate (FDR) *q*-value for that tissue. Non-significant FDR *q*-values (>0.05) are grey. The blue edges signify that the connected nodes have a significant overlap of genes represented.

Gene enrichment analysis of the tissue-specific age-invariant genes revealed a large number of statistically significant GO biological pathway terms (Supplementary Figure 6). As expected, the most enriched terms were largely involved in basic metabolic and structural processes. We also noted many enriched terms were related to the hallmarks of aging [38], which was surprising considering that hallmarks of aging are typically thought to involve processes that change with age (Figure 4A). As an initial step to systematically assess the presence of stably transcribed genes in these hallmarks, we compared the enrichment scores of our tissue age-invariant gene lists with previously published enrichment terms associated with age dysregulation and disease [2, 39].

We first compared our enrichment scores with the top terms recently reported to be associated with mouse transcriptome aging clusters, each displaying a different trajectory with aging and each linked to a different hallmark of aging (Figure 4A) [2]. The top enrichments of these 10 clusters, obtained from the same dataset we performed our discovery on, are associated with hallmarks of aging like protein folding, inflammation, and mitochondrial function [2]. We found our tissue gene sets were significantly enriched in many, but not all, of the clusters. Of note is cluster 3, linked to mitochondrial dysfunction, where age-invariant genes are highly enriched for every term of this cluster. Age-invariant genes are also heavily represented in stress response (cluster 5), signaling (cluster 2), and protein stability (cluster 7). Interestingly, within the protein stability cluster, age-invariant genes were enriched in terms involved in protein folding, processing, and stabilization but not in terms involved in protein localization. The clusters with the least age-invariant genes were those associated with immune response and extracellular matrix. This suggests that hallmarks themselves, or mechanisms within an aging hallmark, can be separated by the presence or absence of age-invariant genes.

Cluster 1 from Schaum et al. is defined as genes that do not change with age and, as expected, has a large overlap with our tissue age-invariant gene sets. Cluster 1 was defined by having the least amplitude (change with age) and least variability. Interestingly, throughout the 17 tissues, only ~33–40% of our age-invariant genes were in Schaum et al.’s cluster 1. The genes not shared between both methods likely reflect the difference between relatively a stable group of genes identified by hierarchical clustering and individual age-invariant genes identified due to their characteristics (as well as our RG requirement that genes be highly expressed) [2, 40]. In RNA-seq, genes with low expression demonstrate significant technical noise making it difficult to assess true biological variability related to age or other factors, and are often filtered out of differential expression studies [41], so our requirement for high expression is useful for focusing on age-invariant genes.

The other ontology terms we examined came from an analysis of age-related diseases and aging hallmarks (Supplementary Figure 7B). Unlike Schaum et al., who used a completely unsupervised approach, Fraser et al. used genes associated with human age-related diseases in a genome-wide association study to define GO biological pathways related to both disease and at least one aging hallmark [39]. Most hallmarks have at least one GO term enriched for age-invariant genes across most of the tissues analyzed (e.g., “steroid hormone-mediated signaling pathway” in altered intercellular communication; “cellular response to insulin stimulus” and “response to nutrient levels” in deregulated nutrient sensing; “macroautophagy” and “regulation of autophagy” for loss of proteostasis; “reactive oxygen species metabolic process” in mitochondrial dysfunction; and “telomere maintenance” in telomere attrition). On the other hand, virtually no GO term related to cellular senescence and epigenetic alterations had high proportions of stably transcribed genes. Thus, two separate methods of defining gene ontology terms for each aging hallmark lead to the same conclusion that age-invariant genes are enriched in terms associated with some, but not all, aging hallmarks.

To better understand the implications of some of these stable pathways, we used the comprehensive resource of mammalian protein complexes (CORUM) database to perform enrichment analysis (Figure 4B) [42]. Complexes involved in mitochondrial function (respiratory chain complex I and cytochrome c oxidase), stress response and signaling (Regulator-AXIN/LKB1-AMPK complexes), and protein stability (COP9 signalosome, proteasome, Parvulin-associated pre-rRNP, and Chaperonin containing TCP1 Complex) are enriched in age-invariant genes.

Our analyses reveal multiple age-invariant genes within pathways that are either dysregulated with aging (Figure 4) or associated with aging pathologies (Supplementary Figure 7B). Some aging hallmarks seem to contain more age-invariant genes than others. Figure 1D depicts a graphical summary of the aging hallmarks by the average significance of the pathways analyzed in Figure 4 and Supplementary Figure 7B, with loss of proteostasis being the hallmark with most aging-invariant genes. Pathways related to the extracellular matrix, cellular senescence, and epigenetic alterations seem particularly devoid of stably expressed genes. These findings are not due to the high expression requirement for our age-invariant genes, as removing this requirement produced similar results (Supplementary Figure 8).

### Age-invariant gene features

Features of genes that change with age have long been a point of discussion in aging transcriptome research, but little is known about the genes that are able to withstand the effects of time. We tested whether our genes have the opposite features to those described in age-dysregulated transcriptome analyses. The features examined are CpG content, DNA methylation (Supplementary Figures 9 and 10), and gene length (Supplementary Figures 9 and 11), given that these features have been implicated in age-associated transcriptional drift [8, 9].

Lee and colleagues reported that genes with CpG islands (CGI+) are less likely to change with age than genes without CpG islands (CGI-) [9]. Accordingly, we found that the proportion of genes with CpG islands located in their promoters increased with each filter, suggesting RGs are more likely to be CGI+ (Supplementary Figure 9A). The transcripts themselves were not enriched for greater %CG content, suggesting there is biological specificity of the function of these islands versus an overall increase in CG content in the region (Supplementary Figure 11D). We next investigated whether age-invariant genes also showed greater stability in promoter methylation status during *in vitro* passaging or *in vivo* aging using reduced-representation bisulfite sequencing (RRBS) datasets. For mouse embryonic fibroblasts serially passaged into senescence, we found both age-variance (based on our skin tissue-specific notation) (Supplementary Figure 10A), and CGI (Supplementary Figure 10B) status influenced methylation variability. Regardless of age-invariant RG status, CGI+ genes are more stable than CGI- genes (Supplementary Figure 10C). However, this pattern was not observed in mouse tissues, including liver, brain, heart, lung, or WBC (Supplementary Figure 10D).

Stroeger et al. report that median transcript length is associated with age-related change, with longer transcripts tending to be downregulated and shorter transcripts tending to be upregulated with age [8]. Complementing these findings, we found that age-invariant genes tend to be shorter than age-variant genes when comparing median (Supplementary Figure 9B) and minimum (Supplementary Figure 11B) transcript length. However, the opposite is true when comparing either maximum (Supplementary Figure 11A) or Ensembl canonical (Supplementary Figure 11C) transcript length.

## DISCUSSION

Much of aging biology research has focused on changes that occur across the organismal lifespan and the contribution of these changes to age-related mortality, morbidity, and functional decline [1, 38]. Molecular signatures that are robust to aging – specifically, age-invariant genes – have received comparatively little attention. Identifying age-invariant genes allows for further study of why they do not change with age, and provides a complementary view of aging and the stability of biological systems with time. Also, from a practical perspective, because many genes change with age, it is important to identify age-invariant genes for use as reference genes (RGs) for gene expression normalization [13]. By adopting a pipeline for identifying RGs from RNA-seq data, we find that there are, in fact, hundreds to thousands of age-invariant genes per tissue. Figure 3A provides a resource for the community to identify aging tissues where classical RGs remain age-invariant. We do find that many classical RGs are appropriate for use in multiple aging tissues. For example, Gapdh is appropriate in 12 of 17 tissues, while Pgk1 is appropriate in 10 of 17 tissues. However, others are not suitable in most tissues - for example, Cdkn1a/p21 is age-invariant in only 4 of 17 tissues, which is unsurprising given its role in aging and cellular senescence [22]. However, none of the classical RGs are suitable for use in cross-sectional aging studies across all 17 tissues studied (Figure 3A), and some classical tissue-gene pairings (e.g., Gapdh in the liver [43]) are not age-invariant. For cases where classical RGs are not suitable for normalization of RT-qPCR experiments in aging studies, our novel age-invariant genes can be utilized instead (Table 1, Supplementary Table 9), as they are not correlated with age and show low variance across the lifespan.

Simply including older mice in our study and utilizing the standard RG identification pipeline was insufficient at filtering out age-invariant genes. Rather, selecting for age-invariant genes required an additional step of explicitly removing genes that are correlated with age. We also find that the variance in expression of a given gene often changes across life stages. For instance, we identified more genes having high variance in young age than in middle or old ages (Supplementary Figure 4). Although perhaps surprising, this finding is consistent with reports indicating the proportion of genes decreasing in variance with age is greater than those increasing in variance with age [6, 7, 44]. It is possible that younger animals show greater variance related to circadian rhythms, the estrous cycles, sex differences, response to stress, or other adaptive and cyclical factors.

Some limitations and caveats should be considered when utilizing our lists of age-invariant genes to test novel RGs, to assess the appropriateness of classical RGs, or to interrogate the biology of age-invariant genes. First, some of the specific cutoffs we utilized were based on prior work, while others (e.g., exact age correlation cutoff) were based on our best judgment. We provide a complete table of filter results in Supplementary Table 2 in case others wish to utilize different cutoffs in selecting RGs. To ensure the list of genes provided are useful reference genes in normalization strategies, including RT-qPCR and even some RNA-sequencing normalization approaches, we required high transcript expression through Filter 4. Although consistent with normalization transcript identification strategies in RNA-seq, many low-expression age-invariant genes are absent. Thus, our lists report age-stable, high-expression genes only. Next, reference genes may be impacted by biological confounders that should be considered when utilizing our age-invariant gene lists. However, our filters inherently limit the effects of any confounders that contribute variability to gene expression within our mouse cohorts. For example, we analyzed both sexes together, seeking genes that satisfy the RG criteria in mixed sex samples. Thus, any differences between sexes, and any variability or change with age within sexes, are inherently limited. However, it is also worth noting that sex-specific gene expression changes with age are well known, i.e., genes that only have age-related changes in only one sex [16, 45–47]. Thus, there may be other genes not included in our final lists that can be age-invariant in only females or males but not both (sex-specific age-invariant genes), or other genes that are invariant within females and males but are significantly different between sexes. For example, it is possible that some classical genes that failed our filters are still age-invariant within one sex, or even within both sexes but with a significant sex difference. Nevertheless, by utilizing mixed-sex samples, our pipeline identified final gene lists that show minimal variation both within and between sexes, and therefore are appropriate for use in both sexes individually as well as in mixed sex samples.

Our findings are influenced by the technical limitations of RNA-seq [10, 48] and the analytical limitations of high-dimensional data, including subsampling of highly heterogeneous samples, previously described in the literature [10, 48, 49]. However, variance in sample collection, processing, and preparation across these datasets likely compensate for any individual source's batch and degradation bias (e.g., each of the four datasets used employs a different poly-A sample preparation kit). Our final 9 pan-tissue age-invariant genes have been tested individually in 17 tissues and four datasets, totaling 1120 samples, thereby reducing the risk of wrongly identifying a gene as age-invariant. Finally, an important assumption not usually discussed in aging transcriptome literature may influence interpretation in the context of aging: consistent RNA mass. A few studies suggest a decline in total cellular RNA mass with aging [50, 51]. This differs from the reported downward trend of differentially expressed genes with age [3]. Current RNA sequencing analysis techniques use proportional estimates (counts per million, fragments per kilobase of transcript per million, transcripts per million, etc.) to normalize samples in order to compare transcript dynamics across samples. Similarly, RT-qPCR protocols typically rely on standardizing total RNA input. If total RNA mass reduction is a global feature of cellular aging, our age-invariant genes are proportionally stable but may decrease in mass with age. Similarly, a gene identified to be overexpressed in old age may maintain constant molar concentration within a cell or tissue. We recommend readers keep these considerations in mind when interpreting any gene expression study in the context of aging.

The existence and study of age-invariant genes have the potential to provide the field of aging with novel insights. It was interesting to find that age-invariant genes were enriched for some pathways associated with hallmarks or pillars of aging (Figure 4), specifically nutrient sensing, proteostasis, and mitochondrial function. We assessed this through GO and KEGG term enrichment, and our findings should motivate future studies to assess the relationship between age-invariant genes and aging hallmarks using other methods. This is somewhat puzzling given that such hallmarks are defined by changes thought to play putatively causal roles in aging [23, 52]. Indeed, genes that most clearly change with age are enriched in the same hallmarks [2]. It is possible that enrichment in pathways associated with hallmarks of aging may simply reflect the fact that hallmarks of aging are broad and cover much of biology. In that case, it may be necessary to more specifically delineate each hallmark of aging, e.g., perhaps only a subset of nutrient sensing processes should be considered as a hallmark. Also, there is no gold standard list of pathways or terms associated with each hallmark of aging. We attempted to address this by using two separate lists reported by prior studies, but acknowledge this as a limitation of our study. This uncertainty should again motivate the aging field to more precisely define aging hallmarks. However, this broadness and uncertainty concerning the hallmarks of aging would not explain our observation that age-invariant genes are associated with only some hallmarks of aging but not others.

What might be the significance of genes associated with hallmarks of aging that remain stably expressed throughout aging? We note that reference and housekeeping gene literature postulates that continuously and stably expressed genes serve essential cellular and organismal functions [12]. A prior report indicated that essential genes are enriched for pro-longevity functions, as experimental overexpression of essential genes tends to increase lifespan in yeast [53]. We also find that age-invariant genes are present in pathways linked to human age-related diseases (Figure 4A, 4B). Thus, we postulate that the age-invariant genes we identified are essential for life, and organisms may have evolved mechanisms to keep these particular genes stable in the face of pervasive age-related changes in the rest of the pathways or networks associated with hallmarks of aging.

Consistent with this hypothesis, depletion of 7 out of 9 of our pan-tissue age-invariant genes have already been reported to induce cell (1110004F10Rik) or embryonic lethality when completely knocked out (Brk1, Rer1, Psmd4, Reco2, Tomm22, and Fis1) [54–59], according to the Mouse Genome Informatics database (http://www.informatics.jax.org/) or International Mouse Phenotyping Consortium databases (https://www.mousephenotype.org/) databases. The remaining two transcripts, Srp14 and Gemin7, have no reported knockout mouse strain or phenotypes, but we hypothesize their absence would be lethal if absent. Furthermore, these genes are involved in mitochondrial function (Fis1, Rexo2, and Tomm22) and proteostasis (Psmd4, Rer1, and Srp14), consistent with the patterns observed using the full list of tissue-specific reference genes. Rexo2 (RNA exonuclease 2) was recently shown to increase mitochondrial gene transcription, mediate RNA turnover, and enforce promoter specificity in mammalian mitochondrial transcription [58]. Rer1 returns rogue ER-resident proteins or unassembled subunits in the Golgi apparatus back to the endoplasmic reticulum [56]. Little is known about the molecular function of the small acidic protein 1110004F10Rik (also known as Smap) or its human ortholog C11orf58, but given its high stability and requirement for cell survival, this protein may merit further attention [54]. Thus, the stability of these 9 genes may have evolved as a result of these genes being critical for mitochondrial and proteostatic function, and for continued life in the face of age-related deterioration.

Another potential example highlighted here is the age-invariant gene enrichment of protein complexes in the electron transport chain. NADH:ubiquinone oxidoreductase, or Mitochondrial Respiratory Complex I, is the only age-invariant gene-enriched electron transport chain (ETC) complex throughout most tissues (Figure 4B). Although the downregulation of ETC genes is one of the most established transcriptional events in aging [49] and protein Complex I proteins undergo major changes in abundance with age [60], stability in some ETC components is likely required for continued life. This is consistent with Complex I being one of the ETC complexes that can be traced back to the last universal common ancestor of all living organisms [61]. Significant dysregulation of such essential components may be incompatible with life, and evolutionary forces may ensure stability throughout the lifespan. It would be interesting to determine whether further bolstering the expression or stability of such age-invariant genes may be a pro-longevity strategy. Alternatively, given their continuous expression across the lifespan, stable genes may be good pharmacological targets within aging tissues. The putative aging intervention metformin, for example, may benefit from the stable expression of its target, complex I [62].

In contrast, age-invariant genes were not enriched in some hallmarks, including epigenetic alterations, cellular senescence, and the extracellular matrix. Our results suggest that these three are the most vulnerable to aging as not many genes related to these hallmarks resist age-related change. In agreement with this finding, these hallmarks are key targets across many existing longevity interventions, i.e., epigenetic reprogramming, senolytics, and enhancing extracellular matrix homeostasis [63–65]. Considering that age-invariant genes tend to be essential for life, one hypothesis is that early changes in these hallmarks may not be particularly detrimental for the organism and thus lack the selective pressure to evolve stability mechanisms in aging. The cumulative long-term burden of changes, however, may contribute to pathological aging. Alternatively, these variant hallmarks may reflect adaptive processes that evolved to change dynamically with aging for the benefit of the organism.

Future analyses could focus on the processes that maintain the stability of age-invariant genes. Our initial investigations demonstrate that age-invariant genes are enriched in CpG islands, consistent with a previous report that genes with CpG islands are more resistant to age-related dysregulation than those without CpG islands, which are misexpressed during age-related heterochromatin decondensation [9]. However, further analyses are needed to determine whether the resistance to changes in the methylome of CpG-rich promoters was responsible for the stability of gene expression over time. For instance, it should be tested whether increased CpG density contributes to reinforcing a stable epigenetic state. We also found that age-invariant genes tend to be shorter than others, confirming a previous study reported that the longest genes show the greatest degree of downregulation [8]. Further study is needed to better understand the relationship between expression dynamics and transcript length. Of note, classical RGs in general have been reported to exhibit shorter introns and exons, low promoter region conservation, 5’ regions with fewer repeated sequences, low nucleosome formation potential, and a higher SINE to LINE ratio [10, 26]. It will be important to determine if and how these factors may contribute to the stability of age-invariant genes.

It will also be important to determine the translatability of our age-invariant transcripts, both to other organisms as well as to protein expression. In a recent study, 52% of human reference genes were matched to independently analyzed mouse reference gene orthologs [14]. Protein abundance can be inferred from transcriptomic data at the tissue and single-cell level, particularly for genes continuously and stably expressed [66, 67]. These transcripts show a high correlation (~0.7) with their protein product except when variability is introduced by cellular state and microenvironment conditions. Given that age-invariant genes are assumed to be expressed in steady-state, many of these genes may also be age-invariant at the protein level.

In summary, we provide the aging field with lists of tissue-specific age-invariant genes as well as 9 pan-tissue age-invariant genes for use in normalization strategies in murine tissues, e.g., RT-qPCR. Interestingly, age-invariant genes are enriched in ontology terms associated with some, but not all, hallmarks of aging. Biological processes that change with age and those that resist age-related dysregulation are two sides of the same coin, and both will need to be investigated to fully understand aging.

## METHODS

### Data preparation and normalization

Four datasets were utilized in this analysis. The Discovery Dataset (GSE132040) consisted of 17 male and female tissues from mice spanning the 4 major life span stages (Figure 1B). 11 of 17 tissues were validated with three datasets of bulk-RNA tissue data from male mice: GSE167665, GSE111164, and GSE141252. Count tables were obtained from GEO and normalized as described below. Sample preparation and alignment can be found in their respective publications [2, 4, 8]. 5 million counts/sample were set as the count threshold for a sample to be included in normalization and further analysis. In the discovery dataset, hierarchical clustering identified a small number of samples that clustered away from their labeled tissue (Supplementary Figure 1A), and examination of tissue-specific markers confirmed they may be mislabeled and, therefore, were removed from analysis (Supplementary Figure 1B). The number of samples removed per tissue and lifestage can be seen in Supplementary Table 10 and those used in the rest of the analysis in Supplementary Table 11. GEO accession number, tissue type, and life stage counts can be found in Supplementary Table 12 for validation datasets. Here, intestine labels refer to samples from both the large and small intestine; and brain to those from both the cerebellum and the frontal cortex.

RNA-seq normalization is essential for proper downstream analysis of datasets. In this study, we identified our genes with two normalization approaches: TPM and TMM. The original reference gene discovery approach described by Eisenberg and Levanon in 2013 [10], utilized RPKM normalized data. Around the same time, conversations about proper data processing produced Transcript Per Million (TPM), an intra-sample normalization method that approximates relative molar RNA concentration (rmc) [17]. TPM was only incorporated into this RG identification approach in 2019 [27]. Another major strategy for data normalization techniques involves between-sample normalization. To prevent normalization-based artifacts, and given there is no single best normalization approach, the discovery data was normalized with two different approaches: TPM and Trimmed Mean of M (TMM) [31]. TMM, an inter-sample normalization method, generates a normalization factor assuming most genes are not differentially expressed. Therefore, TPM is akin to RT-qPCR due to its similarity with rmc while TMM leverages inter-sample information and is less sensitive to gene outliers. Both performed similarly well at identifying RGs in a recent systematic comparison of normalization methods [28].

TPM normalized data was calculated following the formula:


$$\text{TPM}=\frac{\;\frac{\text{\#reads mapped to transcript}}{\text{transcript length}}}{\text{Sum}\left(\frac{\text{\#reads mapped to transcript}}{\text{transcript length}}\right)}\times 10^{6}$$

Transcript lengths used in the above formula were obtained with EDASeq package’s (version 3.13) getGeneLengthAndGCContent function. TMM was calculated using the calcNormFactors function from the edgeR package (version 3.40.1).

Gene expression plotting and validation data were performed only with TPM normalized data. Plots were generated with ggplot2(version 3.4.0), ggforce (version 0.4.1) and ggdendro (version 0.1.23) and ggbreak (version 0.1.2) [68].

### Gene filtering process

Filters were applied sequentially in R (version 4.2.2) as described in Results. Most mathematical calculations used the *r* base and MatrixStats package (version 0.63.0). The filter criteria were applied sequentially in both TMM and TPM normalized data, separately for each tissue, thus yielding different lists for each tissue. For each filter, *x* is either TMM or TPM, and genes were required to pass the filter for both TMM and TPM. Requirements were defined as follows:

1. Continuous expression: For each gene, non-zero expression in all samples. Determined by eliminating genes with any empty or 0 values.
2. Low variance: For each gene, the standard deviation (SD) of the log2 normalized gene (*x*) expression for all samples (*i*) is less than 1. $\forall i,\;\sigma (\log_{2}(x_{i}))<1$.
3. No exceptional expression/outliers: For each gene, log2 normalized values are within two units of the gene’s mean (removing genes with data points four-fold away from the gene mean). $\forall i,\;|\log_{2}(x_{i})\;-\;\mu (\log_{2}(x))|\;\leq 2$.
4. Medium to high gene expression: For each gene, the log2 normalized expression mean is above the mean of all the genes expressed in the particular tissue $\forall i,\;\mu (\log_{2}(x))\geq \mu (\log_{2}(\text{all}\;\text{genes}))$.
5. Low coefficient of variation (CV): For each gene, the percent coefficient of variation (%CV), the ratio of the standard deviation to the mean, is lower than 20%. $\forall i,\;\sigma (\log_{2}(x_{i}))\;/\;\mu (\log_{2}(x))\;x\;100\leq 20$.
6. No correlation between gene expression and age: For each gene, correlation with age was calculated and genes with a Pearson's correlation *p*-value smaller or equal to 0.05/*n*. WGCNA package (version 1.71) function corAndPvalue was used to obtain correlation coefficients and *p*-values. Because each tissue had a 5% chance of finding an association by chance with a fixed 0.05 *p*-value, a gene present in 17 tissues would have a 58% chance of being erroneously discarded 1-(0.095)^17^. We applied a fractional threshold of a 0.05 *p*-value, where the *p*-value threshold applied was 0.05/*n*, where *n* is the number of tissues in which the gene in question passed filters 1–4.
7. For each gene: %CV ≤20 and Spearman correlation *p*-value = 0.05/*n* in a validation dataset. *n* = number of tissues a given gene is present in at filter criteria 6. This step was applied only to TPM normalized data.

### RNA isolation and cDNA synthesis

Frozen liver and heart tissues were gifts from Prof. Ron Korstanje at The Jackson Laboratories. Groups consisted of 3 samples per age (8 and 18 months) and sex (female and male), except there was only one sample for an 18-month-old female liver. RNA was isolated with RNeasy Plus Mini Kit (Qiagen #74134) with pestle and syringe homogenization. cDNA was generated using Iscript gDNA Clear cDNA Synthesis (Bio-Rad #1725035) and equivalent RNA mass per 20uL reaction (500ng of heart and 1ug of liver). RNA concentrations were determined with a Qubit 4 fluorometer (Thermo Fisher #Q33238) and RNA BR Assay Kit (Thermo Fisher Q10210).

### Expression data and RG stability

RT-qPCR reactions were assembled with equivalent SsoAdvanced Universal SYBR Green Supermix (Bio-Rad #1725272), cDNA, and respective PrimePCR SYBR Green primers (Bio-Rad #10025636, AssayIDs Atp6v1f: qMmuCID0014923, Cdkn1a: qMmuCED0046265, Srp14: qMmuCID0020464, Tbp: qMmuCID0040542, Tfrc: qMmuCID0039655, Tomm22: qMmuCED0046631). RT-qPCR was performed in a CFX96 thermocycler (Bio-Rad). Stability algorithms NormFinder [15], BestKeeper [34], geNorm [35], and delta-CT method [36] were calculated and integrated into RefFinder [37]. All calculations were performed in R. geNorm and BestKeeper were calculated with the ctrlGene package (version 1.0.1) [69], Normfinder algorithm was downloaded from moma.dk, delta-CT method and RefFinder functions were recreated as originally described. Metadata for the samples used can be found in Supplementary Table 13, cycle threshold results in Supplementary Table 14 for the heart, and Supplementary Table 15 for the liver.

### Enrichment gene analysis

Enrichment analysis was performed using gprofiler2’s (0.2.1) gost function. Electronically annotated GO terms were included in the analysis, and a common custom background of genes expressed at least once in every tissue was imputed. Bonferroni correction was used to calculate enrichment significance. Aging hallmark trajectory enrichment terms were obtained from Schaum et al. [2], while GO biological process terms associated with age-related disease and aging hallmarks were obtained from Fraser et al. 2022 [39]. A few GO terms identified by Schaum et al. have been discontinued and are marked as obsolete. These terms were excluded from our analysis. Lastly, the top 20 age-invariant GO (biological process, cellular component, and molecular function), KEGG, and Reactome terms were determined by ranking *p*-values within tissues and taking the lowest 20 gene rank sums across tissues.

For the enrichment maps, all 17 sets of enrichment terms (one per tissue) were used in EnrichmentMap in Cytoscape to generate a consensus network. Different consensus parameters used were used for the CORUM [42] (*P*-value: 0.05, FDR *Q*-value: 0.05, Jaccard Overlap Combined: 0.375, test used: Jaccard Overlap Combined Index, *k* constant = 0.5) and GO: BP terms (*P*-value: 0.01, FDR *Q*-value: 0.01, Jaccard: 0.25, test used: Jaccard Index) networks. AutoAnnotate identified common terms for clusters of interconnected nodes. Each node is a pie chart with each slice colored by the enrichment score of each tissue [70]. Average *p*-values for each aging hallmark can be found in Supplementary Table 16.

### CpG island and methylation variability analysis

Gene CpG island (CGI) status was mapped to the annotated list from Lee et al. [9]. Gene names passing each criterion/filter for each tissue were annotated, and percent positive and negative CGI proportion was calculated. Mean and standard deviation were calculated across tissues for each criterion/filter. Counts and percentages of CGI distributions in tissue lists by filter, the odds ratio, statistical test used, and associated *p*-value are listed in Supplementary Table 17.

Composite multi-tissue murine RRBS data [71] was mapped to the mm9 gtf gencode genome. For mouse embryonic fibroblasts, data alignment was previously described [72]. For both datasets, CpG sites common to at least 10 samples and covered by more than 5 reads were analyzed. The methylation status of the promoter region was estimated by averaging the CpG beta values enclosed within 1kb of the transcription start site. Standard deviation was calculated for the methylation of each promoter.

## Supplementary Materials

Supplementary Figures

Supplementary Tables 1-12, 14, 15 and 17

Supplementary Tables 13 and 16
