# Supplementary material for: Age-invariant genes: multi-tissue identification and characterization of murine reference genes
Source: Aging (Albany NY). 2025 Jan 27;17(1):170–202. doi: 10.18632/aging.206192 (PMC11810070; doi:10.18632/aging.206192)
Supplement: Supplementary Figures [file aging-17-206192-s001.pdf]

SUPPLEMENTARY FIGURES

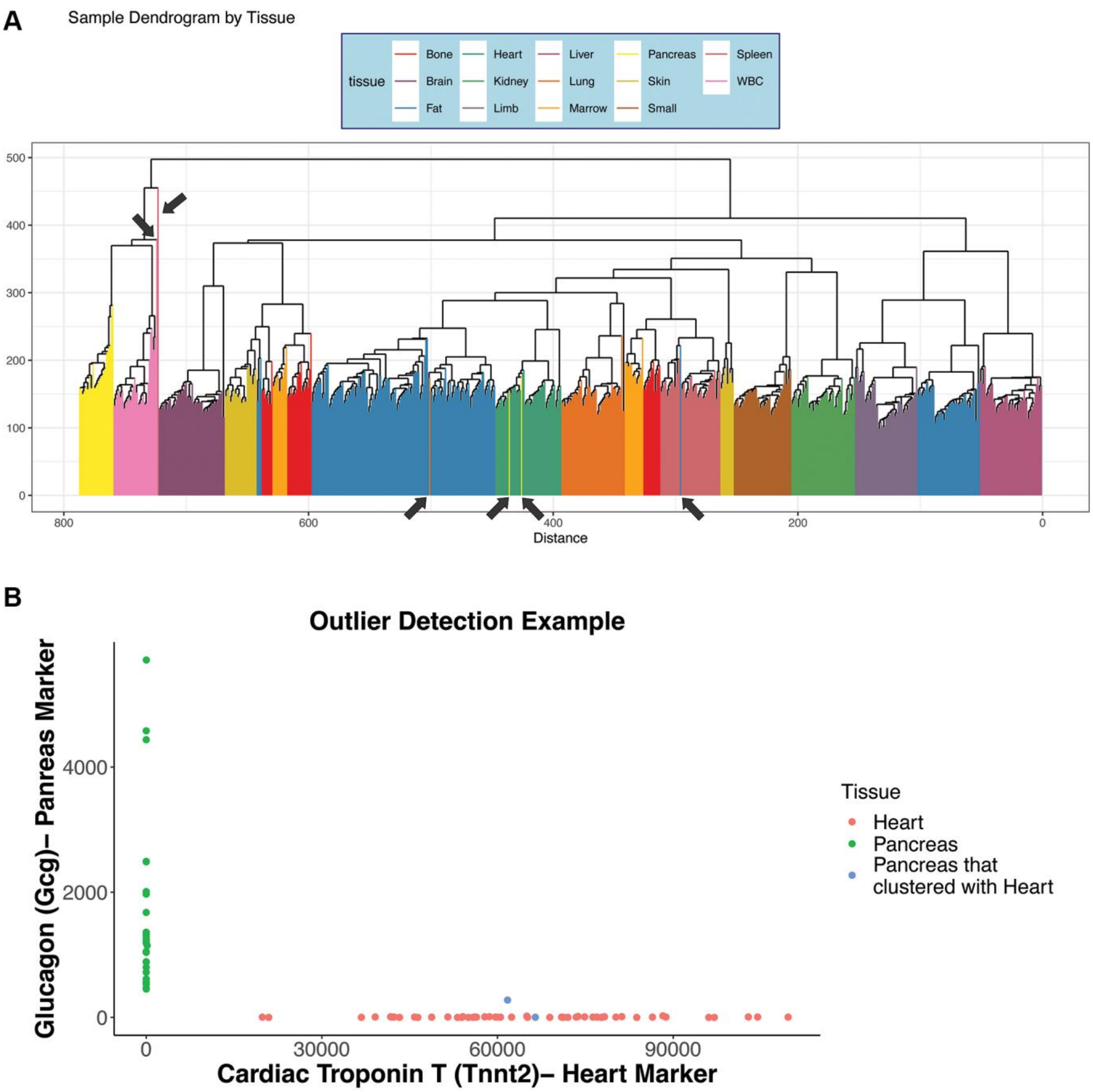

Supplementary Figure 1. Outlier removal strategy. (**A**) Dendrogram of hierarchical clustering of samples above 5 mil reads. Arrows indicate outlier samples that did not cluster with their labeled tissue. (**B**) Example verification of dendrogram outliers. Samples that clustered with other tissues often did not express the labeled tissue's distinctive markers, or expressed markers specific to other tissues.

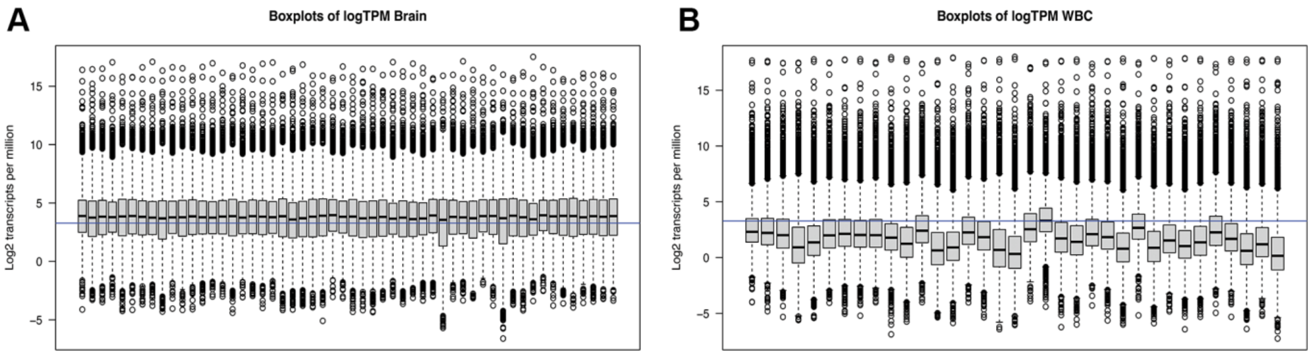

Supplementary Figure 2. Difference in tissue transcript counts. Transcript count distribution (CPM) by sample in Brain and WBCs. Each boxplot on the x-axis represents an individual sample in Brain (**A**) or WBC (**B**). Boxplots represent the 1st quartile, mean and 3rd quartile expression values of all genes in a given tissue in counts per million (CPM).

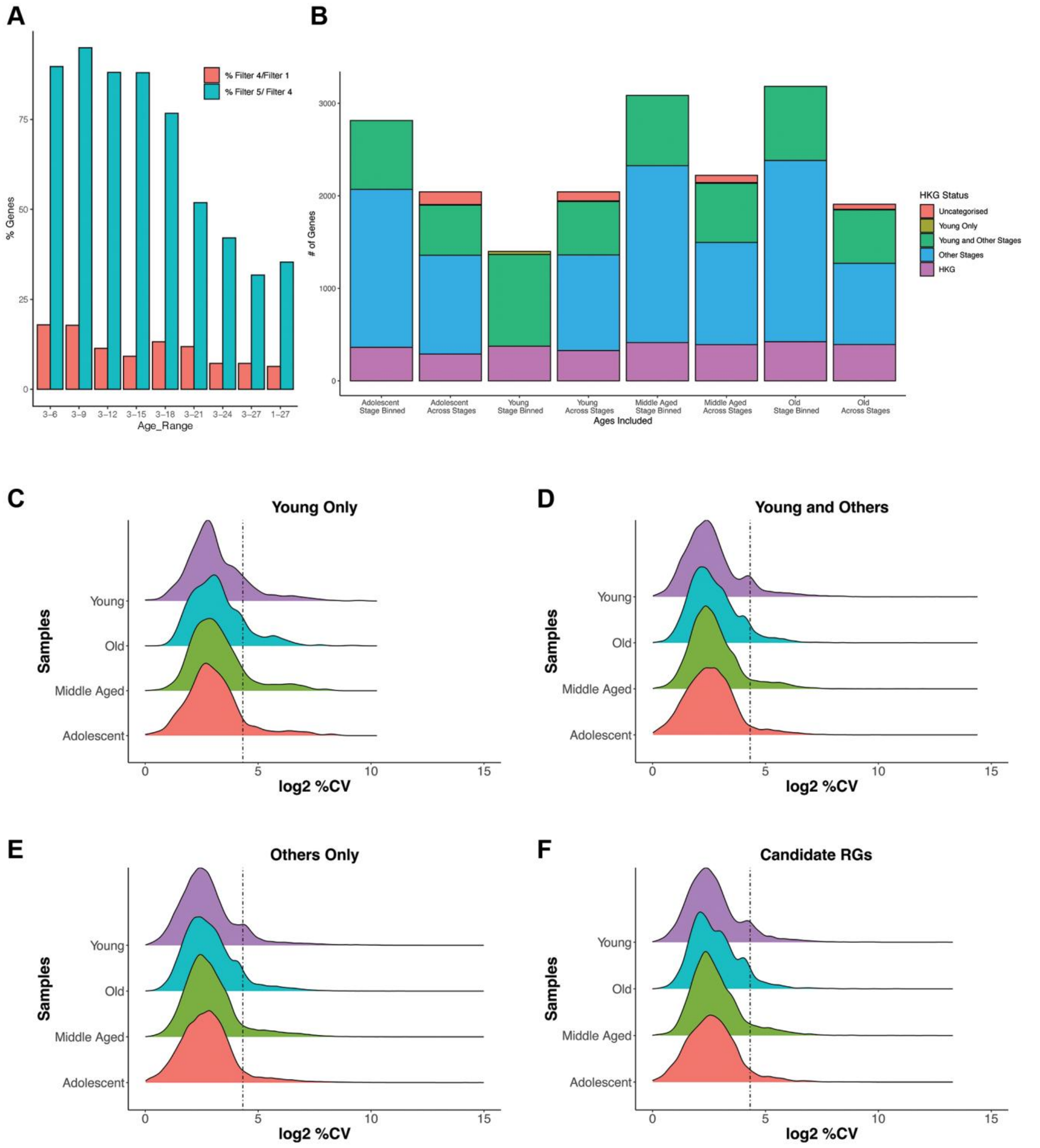

Supplementary Figure 3. Cross-stage sampling improves age-invariable gene identification. (**A**) Adding age groups reduces the number of qualifying age-invariant genes at Filter 4 and Filter 5. %Filter4/Filter1: Percent of qualifying continuously expressed (filter1) genes after filter criteria 2–4. %Filter5/Filter4: Percent of RGs identified using a standard pipeline (filters 1–4) without significant age correlation (Filter 5). (**B**) Number of genes discovered across all tissues (common to all tissues) in a particular lifespan stage (stage binned) or with an equivalent number of random samples (Across Stages). Filter Criteria 1–4 were applied to obtain these results. Across Stages, numbers represent the average of 100 random iterations. Cross-lifespan stage sampling reduces the number of RGs discovered except in Young stage (3 and 6 mo). HKG: Reference Genes (RGs) identified at filter criteria 4 using the whole dataset, Young Only: Genes identified at filter 4 only with the young samples. Young and Other Stages Genes identified separately when performing the analysis with young samples and at least one other lifespan stage. Other Stages: Genes identified except in young lifestage samples. (**C**–**F**) Density plots of the percent coefficient of variance (%CV) in log2 scale of the genes belonging to each of the categories explained in (**B**). %CV was calculated for qualifying genes in each lifespan stage in every tissue. There is a continuous rightward shift, higher variance, in young sample tissues.

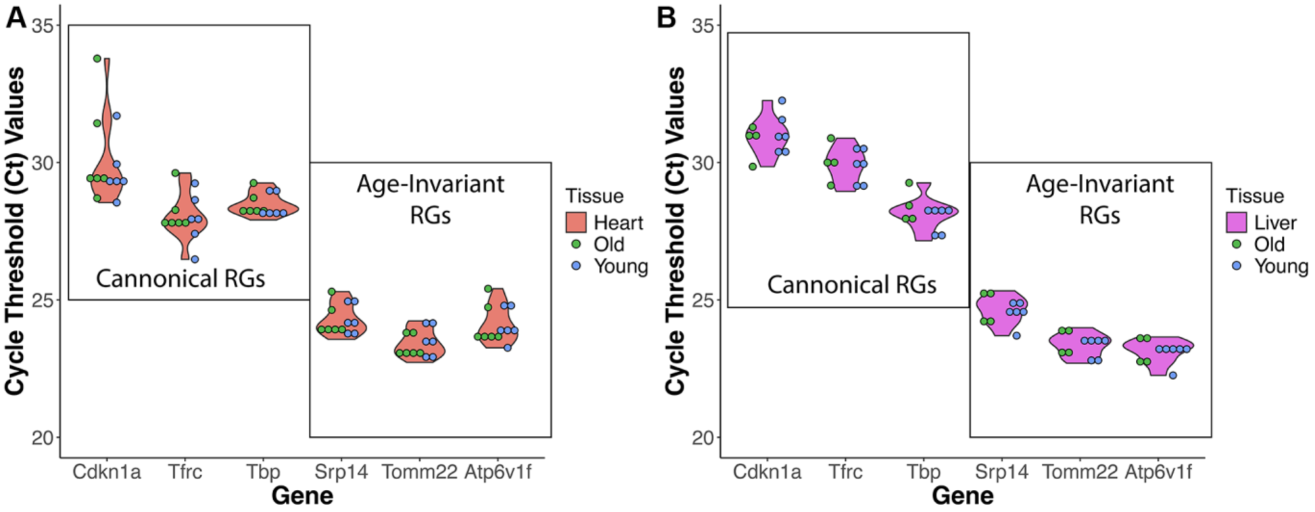

Supplementary Figure 4. RT-qPCR Cycle threshold violin and dot plots of invalid classical (left) and novel age-invariant (right) RGs in Heart (**A**) and Liver (**B**).

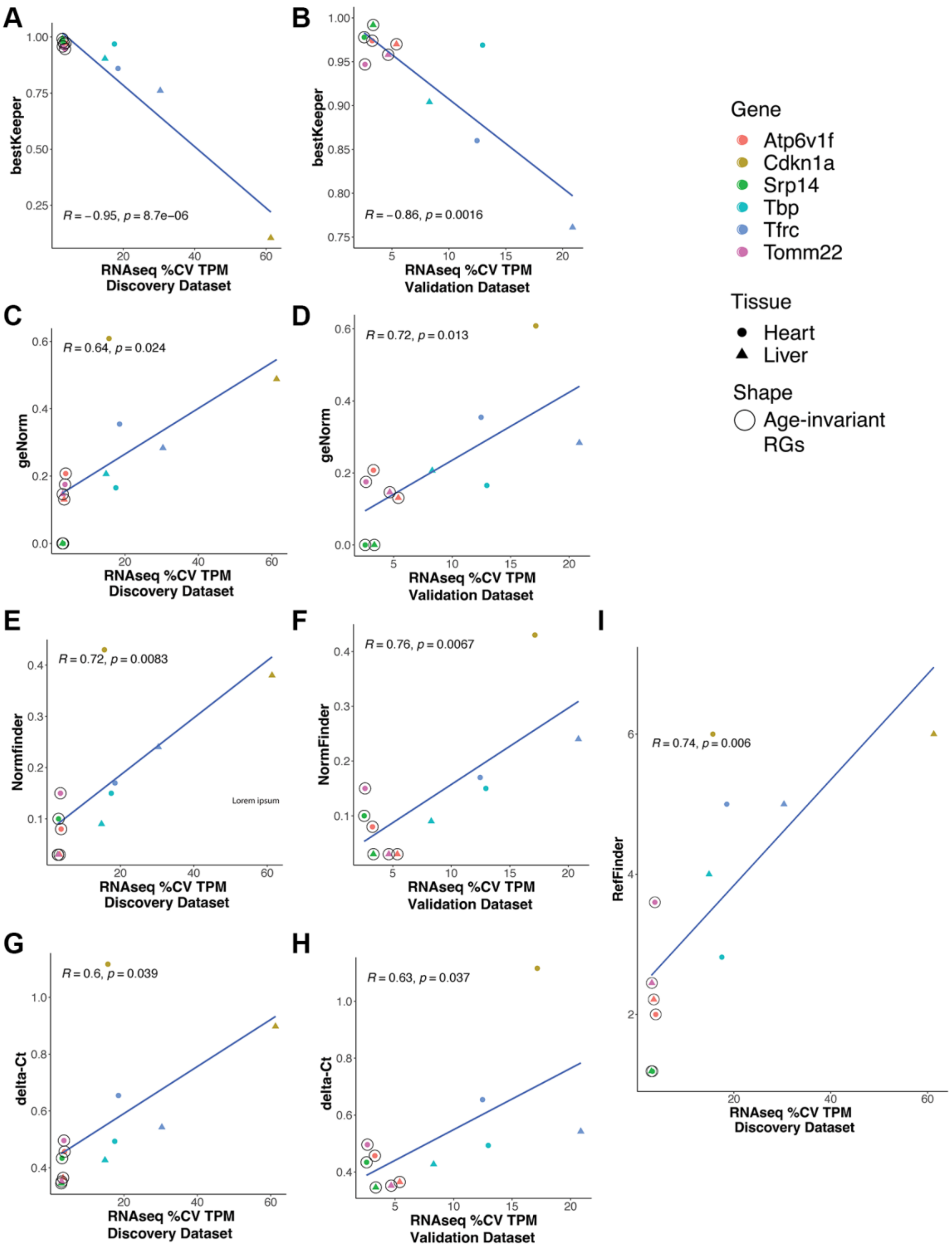

Supplementary Figure 5. Discovered Aging-invariant genes outperform classical RGs in RT-qPCR stability. Plots comparing RT-qPCR stability values: BestKeeper (**A**, **B**), geNorm (**C**, **D**), NormFinder (**E**, **F**), delta-Ct Method (**G**, **H**), and RefFinder (**I**) to the percent Coefficient of Variance (%CV) in the mRNA-seq datasets used to discover (**A**, **C**, **E**, **G**, **I**) and validate (**B**, **D**, **F**, **H**) age-invariant gene lists. Circled points indicate novel age-invariant RGs (Two pan-tissue: Tomm22 and Srp14; and one heart and liver age-invariant gene: Atp6v1f) while uncircled points specify classical RGs from Figure 3A.

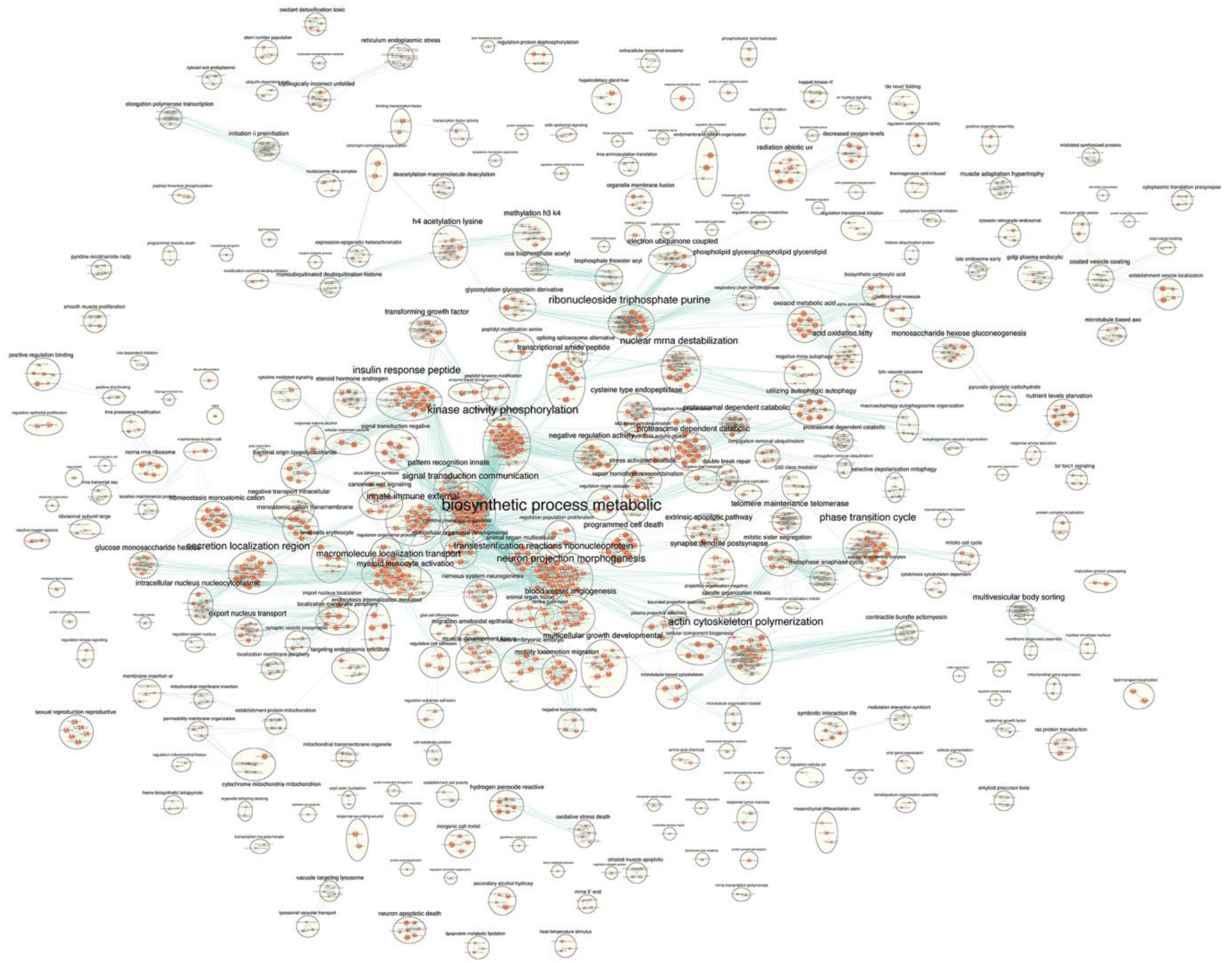

Supplementary Figure 6. GO biological process terms for which age-invariant genes are enriched. Terms wheels follow the same tissue order as Figure 4 and were clustered based on gene overlap. Cluster naming is based on word frequency in the terms included in the cluster. These analyses were performed with Cytoscape.

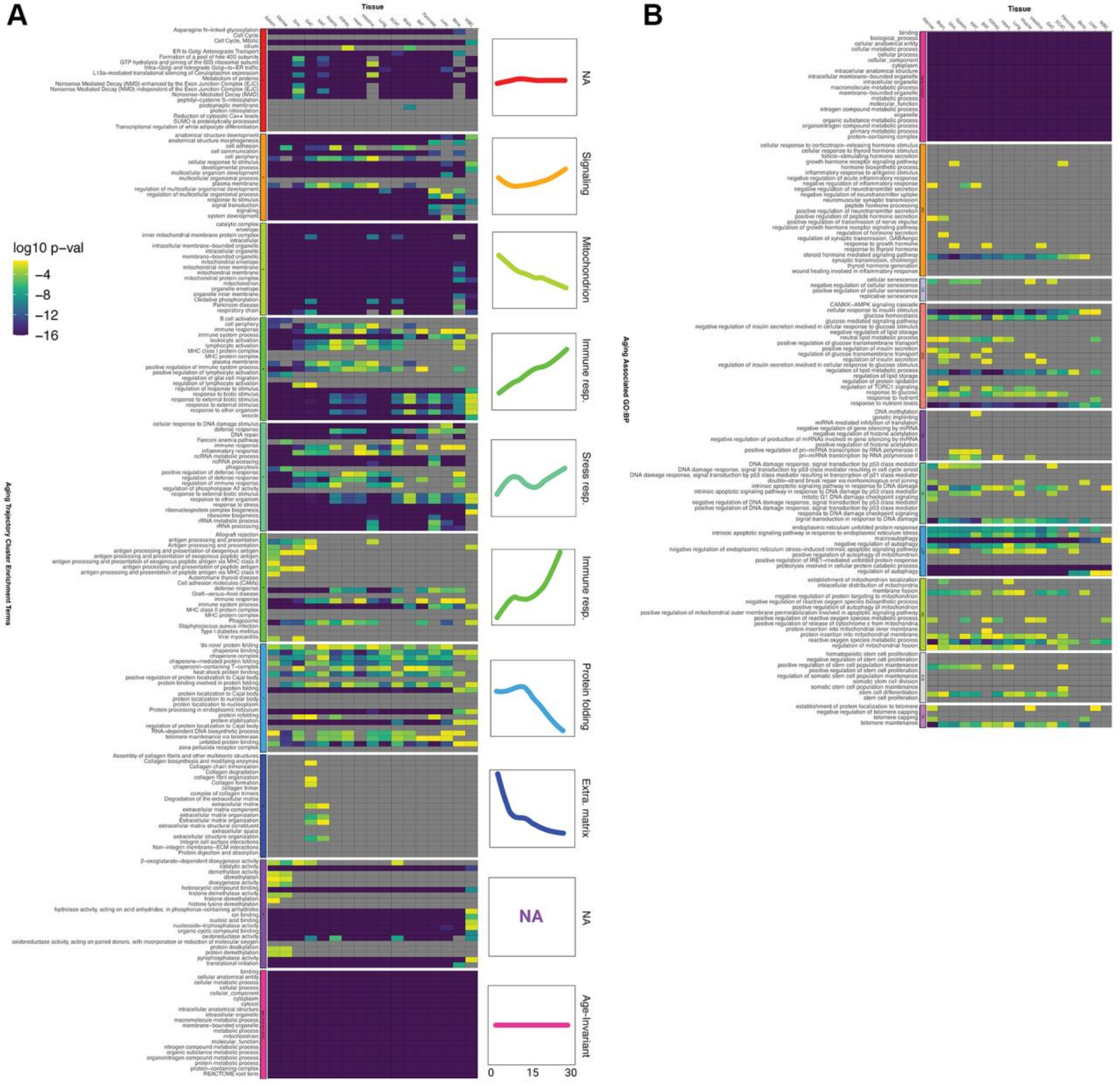

Supplementary Figure 7. Age-invariant genes are enriched for dysregulated and aging disease associated gene functions. (**A**) Tissue age-invariant genes are enriched for some GO, KEGG and REACTOME terms associated with linear and non-linear aging trajectories. Heatmap columns correspond to different tissues, while rows correspond to enrichment terms described in the dataset's original publication (PMCID: PMC7757734) This figure is the same as Figure 4 but with the associated enrichment terms. (**B**) Enrichment of tissue age-invariant genes in terms identified as involved to age-related disease in connection to a hallmark of aging as described in PMID: 9009120.

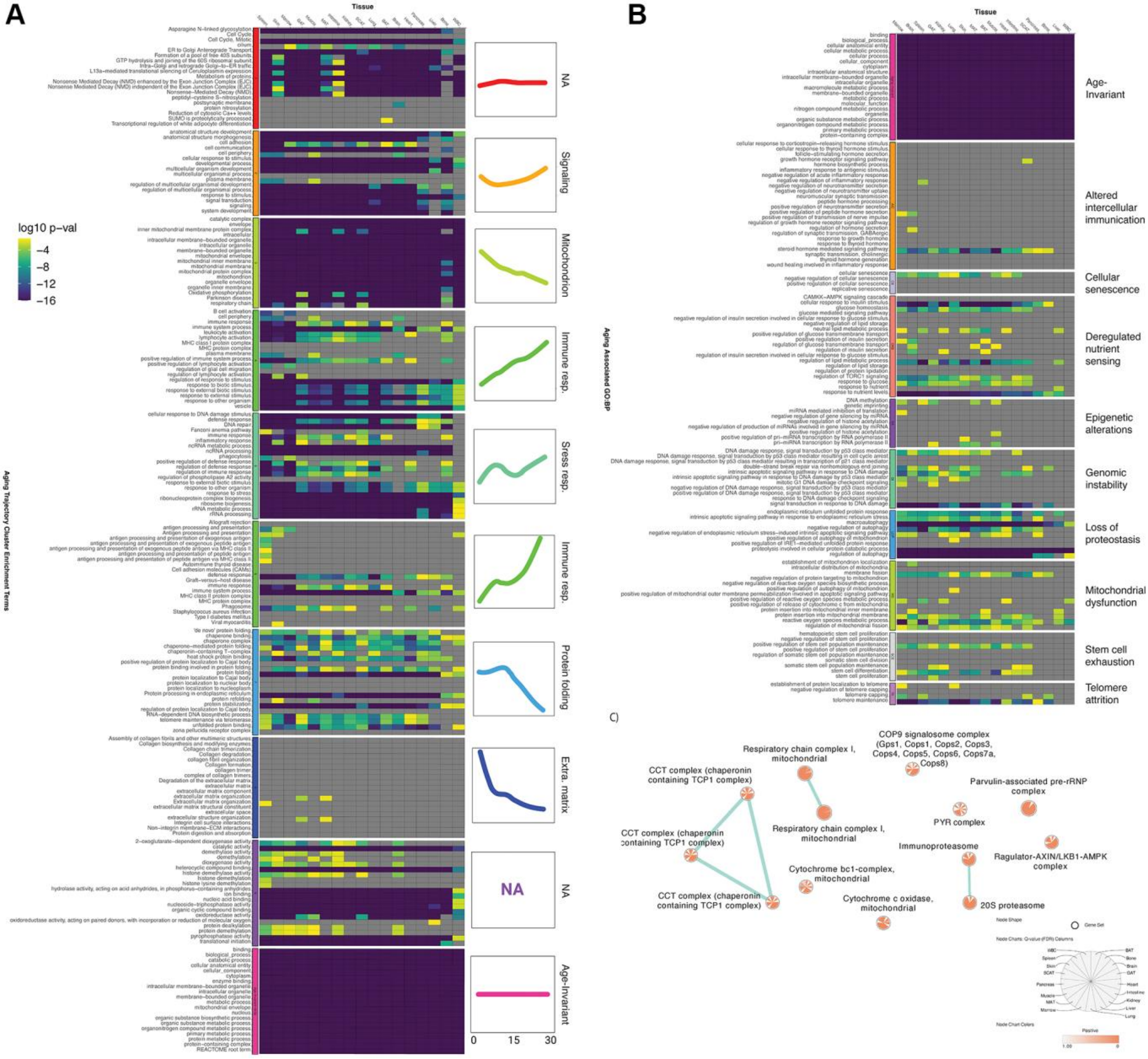

Supplementary Figure 8. Age-invariant genes are enriched for dysregulated and aging disease associated gene functions when removing high-expression requirement. We removed filter4 from the gene selection process to ensure the gene enrichment effect we saw was related to age-invariance rather than high expression. Figures correspond to the original plots. (**A**) Figure displayed in Figure 4A and Supplementary Figure 7A with genes selected without filter 4. (**B**) Figure displayed in Supplementary Figure 7B with genes selected without filter 4. (**C**) Figure displayed in Figure 4B with genes selected without filter 4.

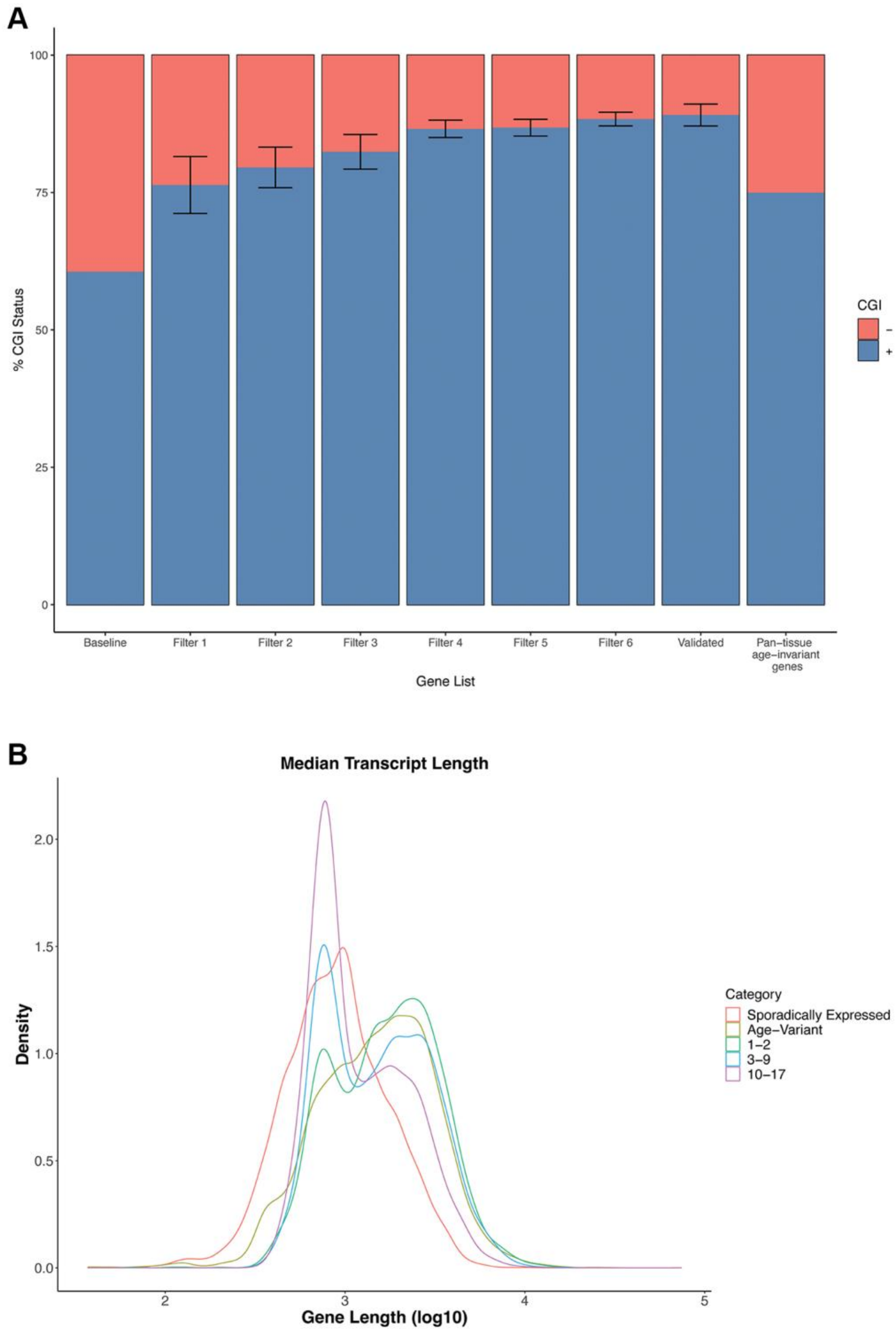

Supplementary Figure 9. Age-invariant gene functions, CpG island (CGI) status, and gene length. (**A**) Proportional distribution (%) of CGI status for genes selected through progressive filters is shown. CGI-positive (CGI+) genes are progressively enriched as age-invariant gene features are selected for. Error bars indicate SD across tissues. The final gene list of pan-tissue genes only includes 9 genes, 2 of which are not CGI+. (**B**) A density plot of the median length of transcript variants associated to each gene in log10. Sporadically and age-variant (but continuously expressed, filter 1) genes are smaller than age-invariant genes. Age-invariant genes are binned according to how many tissues they were identified in.

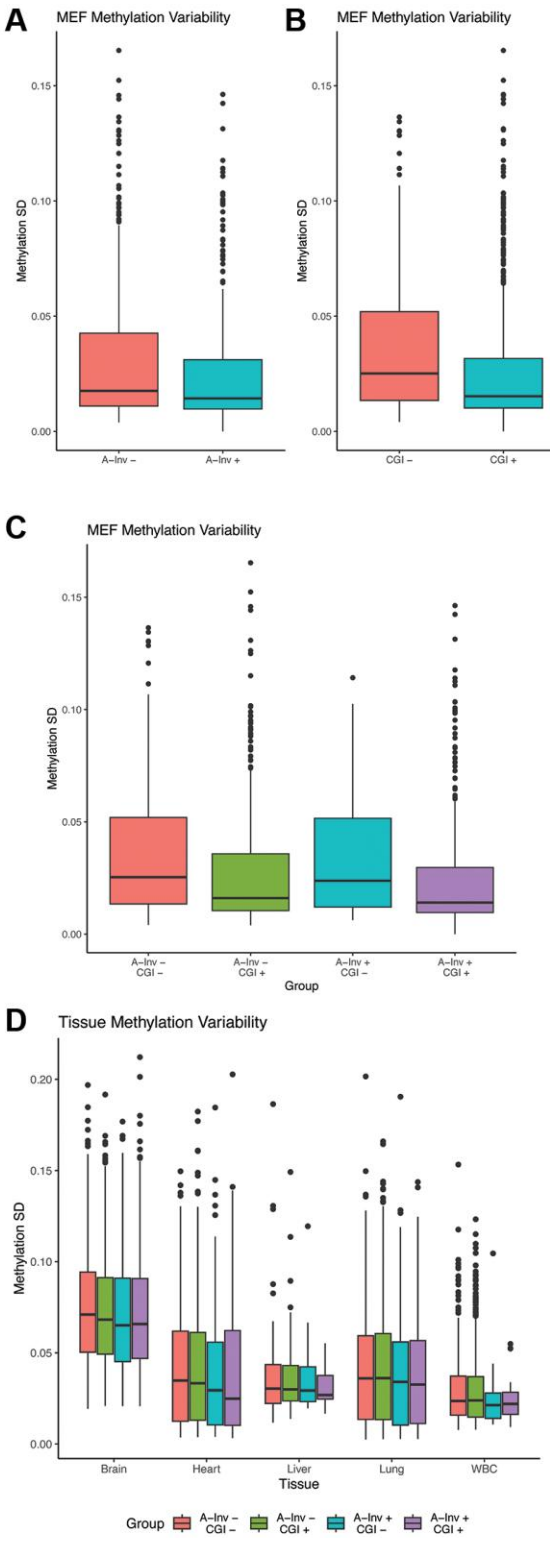

Supplementary Figure 10. *In vitro* and *in vivo* methylation variability in age-variant and age-invariant genes. (**A**–**C**) Methylation standard deviation (SD) values of gene promoter region in serially passaged mouse embryonic fibroblasts (MEF). (**A**) Based on skin age-invariant status, age-variant (A-Inv -) genes have more variable methylation than age-invariant (A-Inv +) genes (*p*-val = 0.02884). (**B**) CpG Island (CGI) status also influences stability, with CGI+ genes being less variable (*p*-val = 4.399e-05). (**C**) Methylation variation in age-invariant genes is driven by CGI status (A-Inv - and CGI - vs. A-Inv - and CGI + *p*-val = 0.0009466, A-Inv - and CGI - vs. A-Inv + and CGI + *p*-val = 5.698e-05). (**D**) Methylation standard deviation (SD) values of tissue age-variant and age-invariant genes in life-stage spanning samples from Brain, Heart, Liver, Lung or WBC. No apparent methylation relationship is seen with age-variant and CGI status. *P*-values obtained with *t*-test.

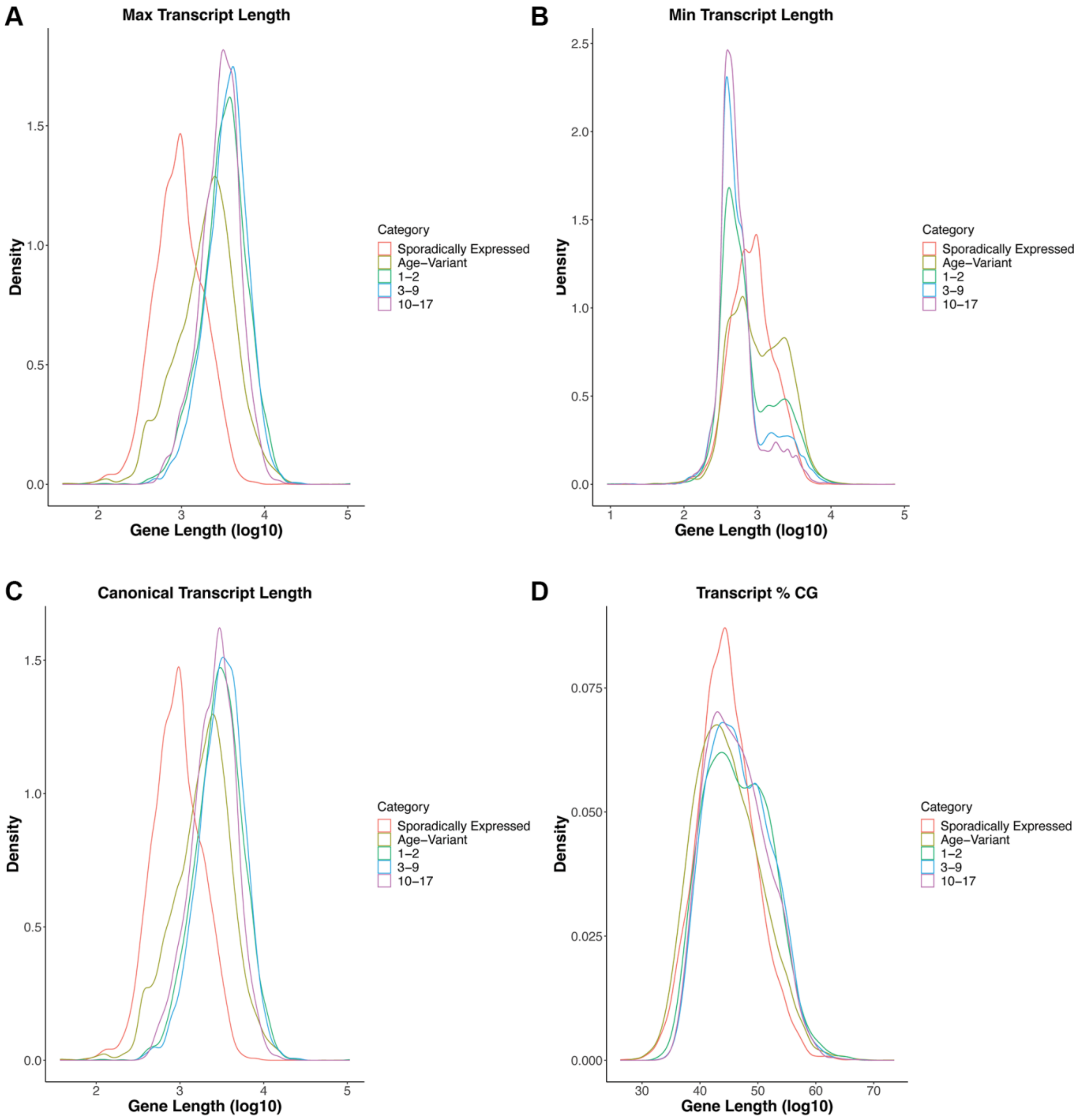

Supplementary Figure 11. Transcript features of age-invariant and age-variant genes. Categories are: sporadically expressed (not continuously expressed), age variant (always expressed but at different levels throughout the data), 1–2 (genes that are age-invariant in one to two tissues), 3–9 (genes that are age invariant in three to nine tissues), and 10–17 (genes that are age-invariant in ten to seventeen tissues). Maximum (**A**), minimum (**B**), and Ensembl canonical (**C**) transcript length of genes. Age-invariant genes have a wide breadth of gene length with larger maximum and smaller minimum transcript lengths. (**D**) %CpG of canonical transcripts. Age-invariant genes have similar CpG content to variant genes.
