# Supplementary material for: Age-invariant genes: multi-tissue identification and characterization of murine reference genes
Source: Aging (Albany NY). 2025 Jan 27;17(1):170–202. doi: 10.18632/aging.206192 (PMC11810070; doi:10.18632/aging.206192)
Supplement: Supplementary Tables 13 and 16 [file aging-17-206192-s003.pdf]

SUPPLEMENTARY TABLES

Supplementary Table 13. Metadata information for the samples used in qPCR analysis.

Name	Sex	Line	Tissue	Birth date	Death/Exit date	Age (weeks)
AgedB6-0278	Female	C57BL/6J	Heart	31-12-2017	24-08-2018	33
AgedB6-0279	Female	C57BL/6J	Heart	31-12-2017	24-08-2018	33
AgedB6-0283	Male	C57BL/6J	Heart	31-12-2017	24-08-2018	33
AgedB6-0284	Male	C57BL/6J	Heart	31-12-2017	24-08-2018	33
AgedB6-0266	Female	C57BL/6J	Heart	09-12-2017	24-08-2018	36
AgedB6-0270	Male	C57BL/6J	Heart	09-12-2017	24-08-2018	36
AgedB6-0215	Male	C57BL/6J	Heart	27-01-2017	24-08-2018	82
AgedB6-0216	Male	C57BL/6J	Heart	27-01-2017	24-08-2018	82
AgedB6-0217	Male	C57BL/6J	Heart	27-01-2017	24-08-2018	82
AgedB6-0206	Female	C57BL/6J	Heart	27-01-2017	24-08-2018	82
AgedB6-0207	Female	C57BL/6J	Heart	27-01-2017	24-08-2018	82
AgedB6-0208	Female	C57BL/6J	Heart	27-01-2017	24-08-2018	82
**Type**	**Harvest Date**	**Line**	**Status**	**Source**	**Age (weeks)**	**Sex**
Liver	24-08-2018	C57BL/6J	Available	AgedB6-0206	82	F
Liver	24-08-2018	C57BL/6J	Available	AgedB6-0207	82	F
Liver	24-08-2018	C57BL/6J	Available	AgedB6-0208	82	F
Liver	24-08-2018	C57BL/6J	Available	AgedB6-0215	82	M
Liver	24-08-2018	C57BL/6J	Available	AgedB6-0216	82	M
Liver	24-08-2018	C57BL/6J	Available	AgedB6-0217	82	M
Liver	24-08-2018	C57BL/6J	Available	AgedB6-0304	35	F
Liver	24-08-2018	C57BL/6J	Available	AgedB6-0278	33	F
Liver	24-08-2018	C57BL/6J	Available	AgedB6-0279	33	F
Liver	24-08-2018	C57BL/6J	Available	AgedB6-0283	33	M
Liver	24-08-2018	C57BL/6J	Available	AgedB6-0303	35	M
Liver	24-08-2018	C57BL/6J	Available	AgedB6-0303	35	M

Supplementary Table 16. Ranked average log10 *p*-value for Aging Hallmark associated GO terms.

Hallmark	Average log10 (*p*-val)
EA	−0.213781602
CS	−0.411091613
AIC	−0.41500978
SCE	−0.645708596
MD	−1.387717035
GI	−1.475028755
TA	−1.758950891
DNS	−2.573837215
LOP	−19.6887982
Aging-invariant	−302.2073638
